# Genetically Determined Variation in Lysis Time Variance in the Bacteriophage φX174

**DOI:** 10.1534/g3.115.024075

**Published:** 2016-02-22

**Authors:** Christopher W. Baker, Craig R. Miller, Tanayott Thaweethai, Jeffrey Yuan, Meghan Hollibaugh Baker, Paul Joyce, Daniel M. Weinreich

**Affiliations:** * Department of Ecology and Evolutionary Biology, Brown University, Providence, Rhode Island 02912; † Department of Mathematics, University of Idaho, Moscow, Idaho 83844; ‡ Department of Biological Sciences, University of Idaho, Moscow, Idaho 83844; § Center for Modeling Complex Interactions, University of Idaho, Moscow, Idaho 83844; ** Center for Computational Molecular Biology, Brown University, Providence, Rhode Island 02912

**Keywords:** lysis time, variance, genetics of adaptation, evolutionary theory, life history

## Abstract

Researchers in evolutionary genetics recently have recognized an exciting opportunity in decomposing beneficial mutations into their proximal, mechanistic determinants. The application of methods and concepts from molecular biology and life history theory to studies of lytic bacteriophages (phages) has allowed them to understand how natural selection sees mutations influencing life history. This work motivated the research presented here, in which we explored whether, under consistent experimental conditions, small differences in the genome of bacteriophage φX174 could lead to altered life history phenotypes among a panel of eight genetically distinct clones. We assessed the clones’ phenotypes by applying a novel statistical framework to the results of a serially sampled parallel infection assay, in which we simultaneously inoculated each of a large number of replicate host volumes with ∼1 phage particle. We sequentially plated the volumes over the course of infection and counted the plaques that formed after incubation. These counts served as a proxy for the number of phage particles in a single volume as a function of time. From repeated assays, we inferred significant, genetically determined heterogeneity in lysis time and burst size, including lysis time variance. These findings are interesting in light of the genetic and phenotypic constraints on the single-protein lysis mechanism of φX174. We speculate briefly on the mechanisms underlying our results, and we discuss the potential importance of lysis time variance in viral evolution.

A key opportunity in evolutionary genetics is the decomposition of beneficial mutations into their proximal, mechanistic determinants. To date, much of this work has applied methods from molecular biology to dissect the basis of protein evolution (Harms and Thornton 2013; Dean and Thornton 2007), but life history theory (Stearns 1992; Roff 2002) similarly decomposes population growth rate into constituent life history traits. It can thus predict which mutations influencing life history will be favored by natural selection (*i.e.*, increase population growth rate) (Stearns *et al.* 2000). The simple life history of the lytic bacteriophage (phage) makes it particularly well suited to this sort of work. Each generation begins with a free phage adsorbing to a host cell and injecting its genome. Transcription and translation facilitate the assembly of progeny phages within the host, and, finally, host cell lysis releases the next generation of free phages. Assuming the density of host cells is not limiting, Wang *et al.* 1996 provided an expression for phage growth rate in terms of mean burst size and mean time to lysis. Later, Bull *et al.* 2006 extended this treatment by incorporating adsorption and phage death rates. Other studies have further developed this formalism (Rabinovitch *et al.* 1999, 2002), with many applying it to explore the mechanistic basis of beneficial mutations in lytic phages (Pepin *et al.* 2006; Shao and Wang 2008; Bull *et al.* 2011). Several have demonstrated that a mutation’s fixation probability depends critically on which component of life history it affects (Wahl and DeHaan 2004; Hubbarde *et al.* 2007; Patwa and Wahl 2008, 2009).

At steady state (*i.e.*, when host cell infections are at their stable age-of-infection distribution), population growth rate is entirely determined by mean life history traits (Bull *et al.* 2011). But the moment a novel mutation appears, it establishes a lineage that, by definition, is far from this equilibrium. In populations deviating from a stable age distribution, variance in both lysis time and adsorption rate may play a significant role in determining growth rate (Storms and Sauvageau 2014), since it may affect how long the deviation from stable age distribution persists (Bull *et al.* 2011).

These findings motivate interest in the possibility of genetic control of higher moments in phage life history traits. Previously, Dennehy and Wang 2011 used a microscopy-based approach to demonstrate heritable variation in lysis time mean and variance among a panel of phage λ holin protein mutants.

To explore heritable variation in phage life history traits, including lysis time variance, we studied a panel of eight genetically distinct clones of the lytic bacteriophage φX174. φX174 is an ubiquitous (Afshinnekoo *et al.* 2015) and industrially important phage (Labrie *et al.* 2014). It was chosen for this research in large part due to its small, well-characterized genome (Hayashi *et al.* 1988), the first of its kind ever sequenced (Sanger *et al.* 1977). In addition, it infects a well-studied host (*Escherichia coli* C), has a short generation time, and is robust to a wide range of temperatures, making it amenable to evolution experiments and other lab manipulations (Wichman and Brown 2010).

The gold standard for measuring phage lysis time and burst size is the one-step phage growth assay (Hyman and Abedon 2009). However, this method affords only limited access to information about lysis time variance and the relationship between burst size and time for a given phage. Therefore, the panel of eight clones was assessed using an updated version of a venerable experimental method first proposed by Burnet 1929, and later extended by Delbrück 1945. We simultaneously inoculated each of a large number of replicate host cell volumes with, on average, less than one phage particle of identical haplotype. These volumes were then plated at 15-sec intervals on lawns of host cells, and the resulting plaques were counted after incubation.

From this assay, we obtained data that could help determine mean and variance in both lysis time and burst size (Figure 1). However, the information we wanted was obscured by a suite of hidden variables. In wells showing lysis events, we did not know when lysis *actually* occurred. We also did not know the number of phage particles originally in these wells and, if that number was greater than one, how many had lysed their hosts. To infer lysis time and burst size from the two observed metrics (time of sampling and plaque count), we somehow had to circumvent these hidden variables. We overcame this challenge by developing a novel inferential apparatus that complemented the experimental framework with a maximum likelihood-based approach.

**Figure 1:**
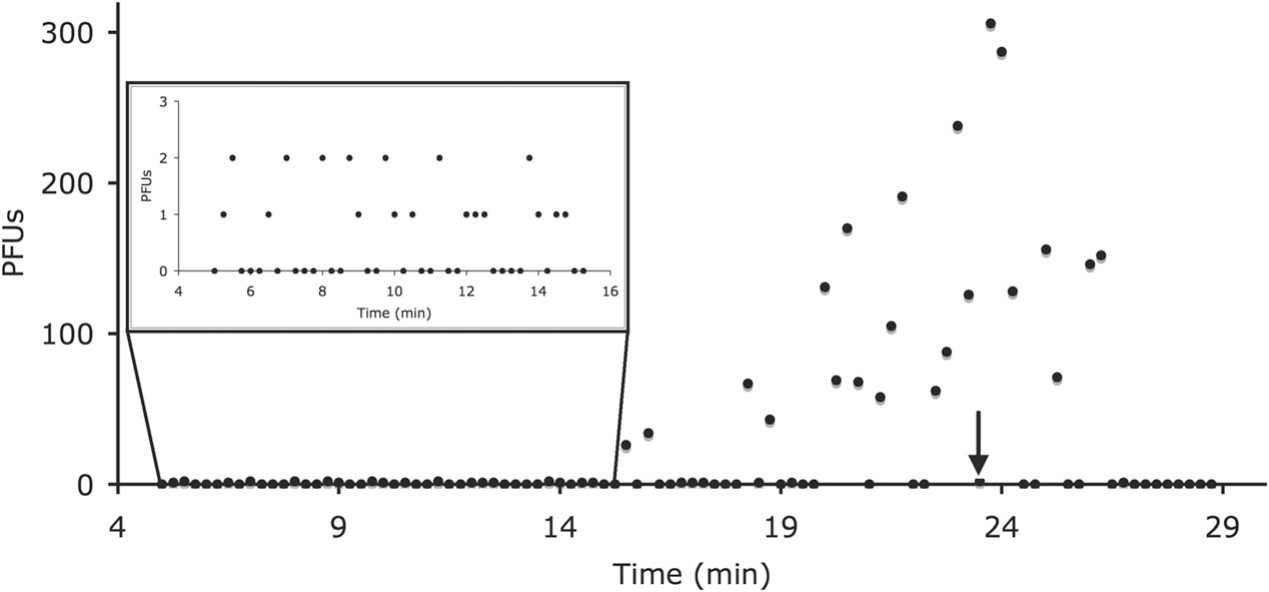
Typical serially sampled parallel infection assay data (*pos5*, ${\text{β}}_{a}$ = 0.71). Here, sampling was performed at 15-sec intervals between 5 min and 28.75 min. At each time point, three outcomes were possible: zero plaques, a “few” plaques (here, fewer than eight; see section *Statistical methods*), and more than a few. Assuming 100% plating efficiency, these outcomes implied respectively that no phage particles were added to the corresponding host cell volume; that the visible number of phage particles was added, but none lysed its host by the time of sampling; and that at least one infected cell had been lysed by the time of sampling. In these data, the first sample with more than a few plaques occurred at 15.5 min, giving an upper bound for the time to lysis ($t_{0}$). Inset illustrates plaque counts prior to the first observed lysis event. The absence of corresponding stratification among plaque counts after 15.5 min implied high variance in burst size. Similarly, the two plaques observed at 23.5 min (arrow) corresponded to two phage particles that had not yet lysed their hosts by that time, implying high variance in lysis time. Thus, these data contain information about higher moments in both burst size and lysis time.

In this article, we describe our finding that, under the tested conditions, genetic heterogeneity could give rise to significant heterogeneity in φX174 lysis time and burst size, including lysis time variance. These findings are interesting in light of the genetic and phenotypic constraints on the single-protein lysis mechanism of φX174, and they point to promising avenues for future theoretical and experimental work.

## Materials and Methods

### Media, host cells, and bacteriophages

Difco LB Lenox Broth (Detroit, MI), pH 7.50, and supplemented with 2 mM CaCl_2_ was used throughout. We studied the performance of φX174 on *E. coli* C [DSMZ 13127, kindly provided by Olivier Tenaillion, Institut National de la Santé et de la Recherche Médicale (INSERM), University Denis Diderot Paris 7], the phage’s natural host. A stock of *E. coli* C was stored in 40% glycerol at –80°. We used this primary stock to seed bacterial colonies on agar plates stored at 4°. Colony plates were created anew every 2–4 wk. To generate experimental bacterial cultures, 10 mL LB was inoculated with a single colony and shaken in a 50-mL flask at 200 rpm and 37° for about 16 hr. The resulting stationary-phase cells were then diluted somewhere between 33- and 80-fold into 40 mL LB in a 250-mL flask. The diluted culture was shaken at 37° for about 3 hr until it reached an optical density $\left(OD_{600}\right)$ of 0.6. Exponential-phase cells prepared in this way were used for each assay.

To synchronize phage infections, we treated cells using a starvation protocol adapted from Denhardt and Sinsheimer 1965 and kindly provided by Darin Rokyta, Florida State University. The treatment starved cells so that they “shut down,” allowing for phage adsorption but not entry. (It is likely that the treatment also had the fortunate side effect of reducing variance in time to cell division compared to an exponential-phase culture, since it was bound to cause a lag in the cell cycle. See *Discussion* for the cell cycle’s relevance.) First, we transferred 1 mL cells to a 1.5-mL centrifuge tube and pelleted them by spinning in an Eppendorf centrifuge (5417R) for 2 min at 20,800 rcf. After aspirating the supernatant, we resuspended the cells in 1 mL HFB-1 solution [0.06 M NH_4_Cl/0.09 M NaCl/0.1 M KCl/0.1 M Tris-HCl (pH 7.4) /1.0 mM MgSO_4_/1.0 mM CaCl_2_] by vortexing for 8 sec. Then, we centrifuged the solution, removed the supernatant, and resuspended in HFB-1 twice more. After removing the supernatant from the third and final HFB-1 rinse, we resuspended cells in 1 ml HFB-2 solution (HFB-1/10 mM MgCl_2_/5 mM CaCl_2_). This served as our culture of starved bacterial cells, ready for phage adsorption.

**Figure 2:**
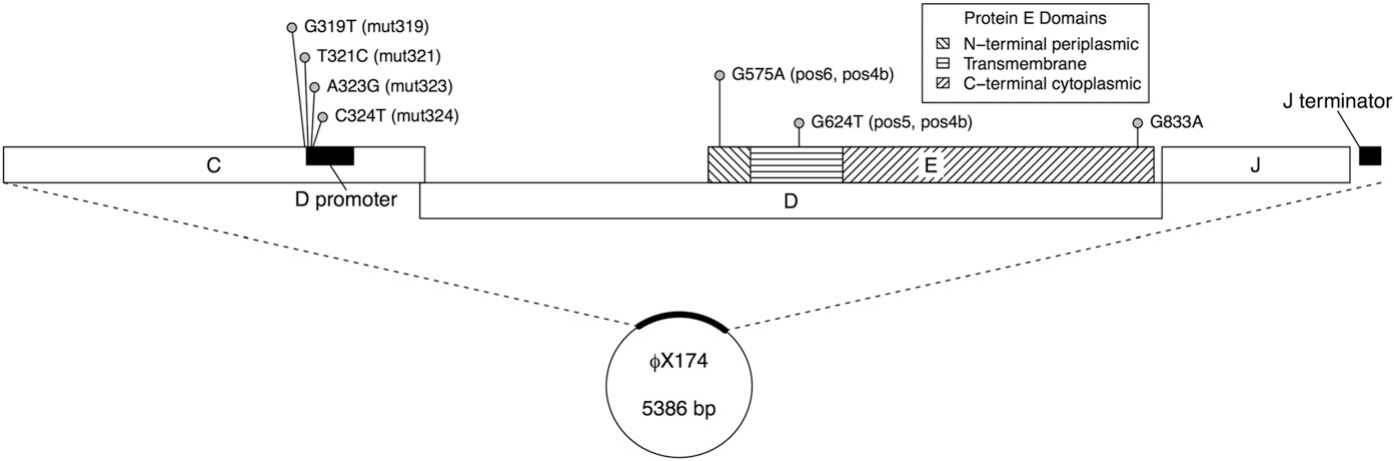
Positions of mutations in φX174 genome. The figure shows a 832-bp region of interest, including genes *C*, *D*, *E*, and *J*. Gene *C* is involved in DNA replication, gene *D* is an external scaffolding protein required for procapsid morphogenesis, gene *E* is responsible for lysis of the host, and gene *J* is required for DNA packaging (Fane *et al.* 2006). Gene *E* is shown in three sections, corresponding to three domains of the protein. The transmembrane domain binds the E protein’s substrate, MraY (Mendel *et al.* 2006; Zheng *et al.* 2009). Mutations are indicated with pins at the position, and the site and nucleotide change are indicated to the right of the pin head. The clone(s) carrying each mutation is indicated in parentheses. Note that one of the five background nucleotide differences between the *Epos* and *D*-promoter mutants (G833A) is included in this figure.

We studied a panel of eight genetically distinct clones, chosen based on reports of lysis profiles different from the wild type. These included three *Epos* mutants carrying point mutations in the *E* gene, whose product is the chief player in φX174 lysis (*pos4b*, *pos5*, and *pos6*, first described in Bernhardt *et al.* 2002; kindly provided by Ry Young and Rohit Kongari, Texas A&M University); four carrying point mutations in the *D*-promoter region, which exerts regulatory control over transcription of the *E* gene (*mut319*, *mut321*, *mut323*, and *mut324*, previously described in Brown *et al.* 2010 and Brown *et al.* 2013; kindly provided by Celeste Brown and Amber Stancik, University of Idaho); and a wild type (also provided by Ry Young and Rohit Kongari) (Table 1 and Figure 2). Clones are available from D.M.W. on request. Presence of the mutations of interest was verified by Sanger sequencing of the 65–730 base region of each clone’s genome (nucleotides numbered as in Sanger *et al.* 1977). The clones’ sequences in this region (GenBank accession KU646482–KU646588) were identical to the wild-type sequence (GenBank accession J02482.1) apart from the noted mutations. The wild type employed was the ancestor of the *Epos* mutants, which differed from the wild-type ancestor of the *D*-promoter mutants (GenBank accession AF176034) at five sites: G833A (nonsynonymous), G1650A (nonsynonymous), T2811C (synonymous), A4518G (nonsynonymous), and T4784C (synonymous) (Pearson *et al.* 1997). The G833A difference entailed an amino acid change in the *E* (but not the *D*) gene, though it fell well outside the region coding for the E protein’s essential transmembrane region (Figure 2) (Tanaka and Clemons Jr 2012).

**Table 1:** Characteristics of **φ**X174 *D*-promoter and *Epos* mutants

Type	Clone	Base Change(s)	Amino Acid Change(s)	Lysis Kinetics Compared to Ancestral WT	
				Lysis Time on *E. coli* C (37°)	Via Transfected Plasmid
*D*-Promoter	*mut319*	G319T*^a^*	V63F	+2 min	N/A
	*mut321*	T321C	None	+2 min	N/A
	*mut323*	A323G	N64G	+0–2 min	N/A
	*mut324*	C324T	None	+0–2 min	N/A
*Epos*	*pos5*	G624T	L19F	+10 min	Slightly faster
	*pos6*	G575A	R3H	Same	Much faster
	*pos4B*	G575A, G624T	R3H, L19F	Same	Much faster

All nonsynonymous *D*-promoter changes are within the *C* gene (Brown *et al.* 2010, 2013). L19F is an amino acid change in the transmembrane region of the E protein, while R3H is a change in E’s periplasmic N-terminus. The same mutation that changes leucine to phenylalanine in E confers a A79S change in the D protein. The five background nucleotide differences between the *D*-promoter and *Epos* mutants are not included in this table (see *Materials and methods*). The rightmost column refers to the bulk lysis kinetics of the mutated *E* genes after they had been cloned onto plasmids and inserted into *E. coli* K-12 $slyD^{+}$ cells (Bernhardt *et al.* 2002). Relevant accession numbers are provided in *Materials and methods*.

a A change adjacent to the *D* promoter’s sigma factor binding site. The three other *D* promoter changes are within the binding site.

Phage titers were determined by combining a known volume of phage stock with 200 μL exponentially growing host cells (described above), and plating in top agar held at 42°, consisting of 7.0 g/L Difco BactoAgar (Detroit, MI) added to LB medium (as described above). After several minutes on the bench at room temperature, we incubated plates agar-side-up at 37°. Preliminary results (not shown) demonstrated that accurate plaque counts required at least 4 hr of incubation. Occasionally, for logistical reasons, we interrupted incubation with overnight refrigeration at 4°.

### Serially sampled parallel infection assay

We began by adding 90 μL starved cells to each well of a TempPlate nonskirted 96-well PCR plate (USA Scientific item no. 1402-9598) at 15°. The PCR plate was mounted on a thermocycler to control its temperature precisely. Using a 12-channel pipettor, we then added 10 μl 30-PFUs/mL phage dilution to each well in each row of 12 wells, allowing for a 30-sec interval between rows. PCR plates were held at 15° for at least 20 min, during which time phages could adsorb to the starved cells. We estimate that 10–20% of phage particles adsorbed to hosts during this incubation period (Celeste Brown, personal communication; Newbold and Sinsheimer 1970).

Then, infections were initiated by raising the PCR plate temperature to 37° and adding 100 μL warm LB (incubated at 37° for at least 45 min) to each well in a row with a 12-channel pipettor, again allowing 30-sec intervals between rows. Consequently, the infective process in each row was staggered by half a minute, allowing ample time for the sampling procedure that followed. Note that the temperature increase alone did not trigger infection. The addition of rich medium, such as LB, was required for infection to advance (Newbold and Sinsheimer 1970). Based on past reports of φX174 attachment kinetics under conditions similar to our assay (Brown *et al.* 2013), unattached phage particles likely adsorbed to hosts very quickly at this time, with an average adsorption time of less than 30 sec [assuming adsorption followed an exponential decay process (Abedon *et al.* 2001)].

It is important to note that, based on our knowledge of the mutated genes’ functions (Fane *et al.* 2006) and past results (Brown *et al.* 2013), the studied mutations likely had little or no effect on phage adsorption rate. This includes the nonsynonymous difference (G1650A) between the *D*-promoter mutants and other clones in coat protein F. While a change in the F protein could theoretically have affected adsorption (Bernal *et al.* 2004), it is unlikely to have done so here, because the difference was merely between one basic polar residue (His) and another (Arg).

Five minutes after infection was initiated, we began sampling wells at 15-sec intervals. Sampling was performed by transferring the entire 200 μL contents of a well to a sterile 96-well PCR plate, held at 4° to halt the infective process. We assume that this sampling procedure did not artificially induce lysis in sampled infections. We staggered sampling between rows such that, factoring in the 30-sec gaps in infection between each row, we sampled one well every 15 sec between *t* = 5 min and *t* = 28.75 min. The endpoint of 28.75 min was chosen on the basis of preliminary experiments that showed most infections ending in lysis by that time. Even though not all phages would lyse their hosts by 28.75 min, we found no evidence that φX174 undergoes T4-like lysis inhibition (Abedon 1992), so we believed that samples taken between 5 min and 28.75 min would give us a reasonably complete picture of the clones’ lysis phenotypes.

At the conclusion of this process, the contents of each sampled well—now at 4°—were plated and incubated, as described, and then counted. Typical results are shown in Figure 1. Four to six replicate assays were performed for each clone.

### Statistical methods

#### The model:

We began with an overview of the model that we assumed generated the data. We then developed methods of estimation from the model and applied them to real experimental data.

Each time an assay was performed, we assumed a small, Poisson-distributed number of phage particles had been added to each well. The mean of the Poisson was allowed to vary between assays, which is to say, we assumed that phage concentrations in the stocks differed by clone and could change over time.

Within the wells, we assumed phage particles adsorbed instantaneously to starved cells upon the initiation of infection. (We discuss the rationale underlying this assumption in the *Results* section). We assumed that each phage particle infected and then lysed a cell *T* minutes after the addition of warm media, where *T* could not be less than a biologically imposed minimum (the latent period), and lysis was equally probable every moment thereafter (though we also considered a model in which lysis probability changed with time, as described below). We assumed that burst size grew linearly with lysis time, a relationship generally assumed and supported by past work (Hutchison and Sinsheimer 1966; Abedon 1989; Wang *et al.* 1996; Abedon *et al.* 2001; Chao *et al.* 2002; Bull *et al.* 2004; Wang 2006; but see Hutchison and Sinsheimer 1963 and Storms *et al.* 2014). The fate of each infecting phage particle within a well was assumed to be independent of other particles in that well. Wells were sampled and plated at 15-sec intervals from *t* = 5 min through *t* = 28.75 min, and we assumed 100% plating efficiency, implying that the plaque count on a plate revealed the true number of phage particles in a well at the time of sampling. A plaque count of zero meant no particles were in the well, a count of one to a few implied that as many particles as visible plaques were added but that no host cells had been lysed yet, and a count of more than a few represented the sum of the burst sizes of lysis events prior to the sample time, plus any infected yet unlysed cells. Note that, in the third case, we did not know how many phage particles were in the well to begin with, we did not know how many lysis events had occurred by the time of sampling, and, of the lysis event(s) that had occurred, we did not know when they occurred, except that they must have occurred prior to the moment of sampling. From a statistical perspective, this suite of hidden variables made the problem both interesting and challenging.

#### Notation:

Developing estimators under this model required substantial notation. Table 2 summarizes this notation for the reader’s reference.

**Table 2:** Summary of notation used in statistical modeling

Symbol	Meaning
$\beta_{a}$	The number of phages in a well from assay *a* is Poisson with mean $\beta_{a}$
*p*	Probability a random phage in a randomly selected well in the experiment has not lysed its host
$L_{a}$	# of wells examined in assay *a*
$R_{a}$	# of wells examined in assay *a* that show lysis events
$M_{a}$	# of wells examined in assay *a* that do not show lysis events but have counts ≥1
$Z_{a}$	# of wells examined in assay *a* that have zero phages in them
$N_{ar}$	# of phages in well *r* from assay *a*. We reorder wells such that $r=1,2,\cdots ,Z_{a}$ have 0 phages, wells $r=Z_{a}+1,Z_{a}+2,\cdots ,Z_{a}+M_{a}$ do not show lysis events (but are nonzero), and $r=Z_{a}+M_{a}+1,Z_{a}+M_{a}+2,\cdots ,L_{a}$ are wells showing lysis events
$\bar{N_{a}}$	Mean count in wells not showing lysis events; $\sum_{r=Z_{a}+1}^{M_{a}}N_{ar}/M_{a}$
$T_{iar}$	Time to lysis of phage *i* in well *r* from assay *a*
$D_{T}$	Burst size of a phage that lysed its host at time *T*
$C_{tar}$	Observed count in well *r* sampled in assay *a* at time *t*
$B_{tar}$	The number of phages in well *r*, assay *a*, that have lysed their hosts by time *t* when well is sampled; $B_{tar}=\sum_{i=1}^{N_{ar}}I\left\{T_{iar}\leq t\right\}$
$Y_{tar}$	An indicator function that is 1 when $B_{tar}>0$ and 0 when $B_{tar}=0$
$U_{tar}$	The number of phages in well *r*, assay *a*, yet to lyse their hosts when sampling occurs at time *t*; $U_{tar}=N_{ar}-B_{tar}$
$X_{tdr}$	Sum of lysis times for all phages in well *r*, assay *a*, that have lysed their hosts prior to sampling at time *t*; $X_{tar}=\sum_{i=1}^{N_{ar}}I\left\{T_{iar}\leq t\right\}$
α, μ, σ	Parameters describing the assumed linear relationship between burst size and time $C_{T}=\mu +\alpha T+\varepsilon$ where $\varepsilon ∼N\left(0,{\text{σ}}^{2}\right)$
$t_{0}$	Latent period. For $t<t_{0}$, the lysis rate = 0; for $t>t_{0}$ it is constant per unit time
$w_{t}$	The probability a phage will have lysed its host by time *t*
λ	Parameter relating lysis probability to time; $w_{t}=1-e^{-\left(t-t_{0}\right)/\lambda }$

#### Number of phage particles per well:

Our first task was to estimate ${\text{β}}_{a}$, the Poisson parameter that governs the probability of having 0,1,2,⋯ phage particles in each well for assay *a*. This was somewhat complicated by the fact that, before the latent period ended and lysis began, we observed the actual Poisson counts, but after this period, we only observed the true Poisson counts for “zero” wells and wells that do not show lysis events. In wells showing lysis events, we knew only that the Poisson count must have been greater than zero. To derive an estimator tailored to the situation, we ignored the time element of the data and defined *p* as the probability that a randomly selected phage particle from a randomly selected well would not have lysed its host. We let *n* be the Poisson number of phage particles initially in a well (*i.e.*, before lysis occurs). Then, we noted that each sampled well fell into one of three categories: samples with zero phage particles, samples with phage particles but no lysis events, and samples showing lysis events. The probability of a well belonging to each category was, respectively, $e^{-{\text{β}}_{a}}$, ${\left(p{\text{β}}_{a}\right)}^{n}e^{-{\text{β}}_{a}}/n!$, and $1-e^{-\left(1-p\right){\text{β}}_{a}}$.

We defined wells not showing lysis events as those having plaque counts greater than zero, and less than or equal to seven. When the Poisson parameter *β* is less than or equal to one, counts greater than seven are highly improbable in wells where lysis has not occurred (P<0.001). Of course, it is possible that wells showing fewer than eight plaques could in fact be the results of infections. But, ultimately, we had to set the threshold for distinguishing lysis events from nonlysis events *somewhere*. This threshold-setting exercise was not straightforward. Complicating factors included the following:

1. Poisson average numbers of particles per well varied for each assay, so setting the threshold based purely on the Poisson probabilities was not trivial.
2. There was a nonzero chance of slight inaccuracies in some plaque counts. Rare errors could have occurred in the counting of plaques that would have increased counts on average (*e.g.*, plaque-like air bubbles). In addition, certain experimental manipulations (*e.g.*, mechanical stress from pipetting) could feasibly have induced very premature lysis of some cells, resulting in plates with extremely low plaque counts that otherwise would have had just one plaque.
3. The few plaque counts of six and seven, a couple of which were sampled at unusually early time points, could have exerted disproportionate influence on estimates of clones’ lysis phenotypes if included as lysis events. This is because, as discussed below, an artificially early lysis event would force the value of the $t_{0}$ parameter to shift to some time before that event.

Therefore, given these intermediate plaque counts’ rarity, their potential origins in experimental error, and their potential to distort our estimates of clones’ lysis phenotypes, we chose to set the threshold for lysis events at greater than or equal to eight. It is worth noting, however, that when our data instead were analyzed using a threshold of greater than or equal to five, our results did not change qualitatively (analysis not shown).

We let the number of wells in assay *a* be $L_{a}$ and the number of wells that showed lysis events be $R_{a}$. The likelihood of the data, then, was

$$L\left(N_{a1},N_{a2},\cdots ,N_{aM_{a}},R_{a}\right)=\frac{\prod_{i=1}^{M_{a}}{\left(p{\text{β}}_{a}\right)}^{N_{ar}}e^{-\beta_{a}}}{N_{ar}!}\cdot \left(\begin{array}{c} L_{a}\\ R_{a} \end{array}\right){\left(1-e^{-\left(1-p\right){\text{β}}_{a}}\right)}^{R_{a}}$$ (1)

We then took the log of the likelihood, the partial derivatives with respect to $\beta_{a}$ and *p*, set each equal to zero, and did a considerable amount of algebra (see Appendix 1 for details). This resulted in the following estimator:

$${\hat{\beta }}_{a}={\bar{N}}_{a}+\ln\left(M_{a}+R_{a}\right)-\ln\left(M_{a}\right)$$ (2)

We validated this estimator by simulating data under $\beta_{a}=0.5,1,1.5,$ and 2, and we compared it to two reasonable alternatives: the “zero-class” estimator [$\hat{\beta }=-\log_{e}\left(p_{zero}\right)$, where $p_{\text{zero}}$ is the proportion of sampled wells that had plaque counts of zero], and the “mean-count” estimator ($\hat{\beta }=\bar{X}$, where *X* is the mean among observed plaque counts, applied only to wells sampled before we witnessed any lysis events). The results (which we will not cover in detail) revealed that Equation 2 had a very slightly positive bias (≈3.5% when $\beta_{a}=0.5$, falling to ≈1.5% at $\beta_{a}=2$) but a lower mean squared error than the other estimators across conditions. Valuing the nearness of the estimate to the truth above all else, we adopted the new estimator of $\beta_{a}$ throughout.

#### Time to lysis:

We considered two nested models describing time to lysis. Both assumed that phages do not lyse cells until the latent period of $t_{0}$ ends. The exponential model assumed that lysis occurred with equal probability each moment after $t_{0}$, while the Weibull model allowed the probability of lysis to change with time. Under the Weibull, the time to lysis, *T*, had the following distribution:

$$P\left(T<t\right)=w_{t}=1-e^{-{\left(\left(t-t_{0}\right)/\lambda \right)}^{\text{α}}}$$ (3)

where α was a hazard parameter that dictated how lysis probability changed with time. When α=1, the Weibull model simplified to the exponential model. Because they are nested, we employed a likelihood ratio test to determine if the improved fit under the Weibull justified the additional parameter. To do so, we analyzed each genetically distinct clone separately. We fitted the data to each model using maximum likelihood (as described below), estimated parameters, obtained the log-likelihood, and took the difference: $LR_{\text{obs}}$. We then simulated 100 datasets under the null (exponential) parameter estimates. For each simulated dataset, we fitted the data to both models and calculated the difference in their log-likelihoods: $LR_{\text{boot}}$. We then approximated the *P*-value by the proportion of $LR_{\text{boot}}$ greater than or equal to $LR_{\text{obs}}$. Large *P*-values (*i.e.*, P>0.05) would indicate the simpler exponential model described the data sufficiently well. This test returned *P*-values greater than 0.3 for all clones, and we therefore assumed the exponential model for the duration of the study. One important implication of this assumption was that, under the exponential model, the lysis time variance was equivalent to ${\text{λ}}^{2}$.

Defining $B_{tar}$ as the number of phages in well *r*, assay *a* that had lysed their hosts by time *t*, $B_{tar}$ followed the Poisson distribution with rate $\beta_{a}w_{t}$. If we let $Y_{\text{tar}}$ be an indicator function that is one when $B_{\text{tar}}$ is greater than or equal to one, and zero when $B_{tar}$ is zero, and we let $U_{\text{tar}}$ be the number of phages that had not lysed their hosts in well *r*, assay *a* at time *t*, we show in Appendix 2 that the log-likelihood of the data were proportional to

$$\begin{array}{c} \ln L\left(\lambda ,t_{0}\right)∼\sum_{a}\sum_{t}\sum_{r}-\left(1-Y_{tar}\right)\beta_{d}w_{t}+Y_{tar}\ln\left(1-e^{-\beta_{d}w_{t}}\right)\\ +\left(1-Y_{tar}\right)\left(U_{tar}\ln\left(\left(1-w_{t}\right)\beta_{d}\right)-\beta_{d}\left(1-w_{t}\right)\right). \end{array}$$ (4)

Note that $\sum_{a}$ directed us to sum over all assays of a particular clone. We analyzed each clone separately. We have omitted a clone subscript throughout to avoid further complicating notation. In practice, we obtained joint maximum likelihood estimates of λ and $t_{0}$ by establishing a grid of joint values at narrow intervals, calculating the log-likelihood of each using Equation 3, and selecting those values that together give the best likelihood. Confidence intervals on λ and $t_{0}$ were obtained by parametric bootstrap. To do this, we used $\beta_{a}$, $\hat{\lambda }$, and ${\hat{t}}_{0}$ to simulate one suite of datasets to match (in number and well count) the real datasets for the clone under analysis. We analyzed the simulated suite of datasets using Equation 4 and a grid of potential values to obtain ${\hat{\lambda }}_{\text{boot}}$ and ${\hat{t}}_{0,boot}$. The one condition we imposed in this estimation step was that ${\hat{t}}_{0,boot}$ must be less than or equal to the minimum observed lysis time from the real data. If we did not do this, the bootstrap would have returned values and hence confidence bounds for $t_{0}$ greater than this minimum lysis time, and we knew such values would not be valid confidence bounds when the probability of the real data under them is zero. We repeated 500 bootstrap replicates. We then sorted the ${\hat{\lambda }}_{boot}$ values, and defined the 95% confidence interval as the central 95% of the values. We independently sorted the ${\hat{t}}_{0,boot}$ values and used the central 95% of these. Thus, the confidence intervals on $\hat{\lambda }$ and ${\hat{t}}_{0}$ were not joint. We did not validate that these intervals capture the truth greater than or equal to 95% of the time, but we did confirm that the means of ${\hat{\lambda }}_{boot}$ and ${\hat{t}}_{0,boot}$ were close to $\hat{\lambda }$ and ${\hat{t}}_{0}$, respectively, suggesting that the parametric bootstrap was a valid technique.

We also were interested in inferring mean lysis times from our data. Given the unique properties of exponential distributions, we could derive each clone’s mean lysis time simply by summing its estimated $t_{0}$ and λ. A confidence interval for this estimate could be obtained by repeating this procedure using the upper and lower bounds on the individual parameter estimates.

We now turn our attention to how to determine whether lysis time parameters λ and $t_{0}$ are different for different clones. We conducted several formal tests for differences between clones using a likelihood ratio framework. To test whether both λ and $t_{0}$ clones differ, we note that, under the null hypothesis, a single shifted exponential function would describe the data from both clones being compared; the alternative hypothesis asserted the data for each clone came from a distinct function. We calculated the log-likelihood of the data under the null ($L_{Null}$) by pooling both clones’ data, fitting as just described to obtain ${\hat{\lambda }}_{Null}$ and ${\hat{t}}_{0\left(Null\right)}$, and then using Equation 4. We calculated the log-likelihood under the alternative ($L_{Alt}$) by doing the same thing, except we fitted each clone’s datasets separately, calculated their log-likelihoods separately, and then summed. We define $\Lambda_{Obs}=L_{Null}-L_{Alt}$. To determine whether ${\text{Λ}}_{\text{Obs}}$ is significantly large, we needed the distribution of Λ under the null. We obtained this by simulation. We simulated data analogous in size and sample times to the pooled data using ${\hat{\lambda }}_{Null}$ and ${\hat{t}}_{0\left(Null\right)}$, repeated the fitting process, and calculated log-likelihoods and their difference to yield $\Lambda_{Sim}$. We repeated this process 500 times, and took the approximate *P*-value of $\Lambda_{Obs}$ as the proportion of bootstrap simulations where $\Lambda_{Sim}$ was greater than $\Lambda_{Obs}$.

To test whether just λ differed between pairs of clones, we used a similar likelihood ratio test. Under the null, both clones shared a common value of λ but could have different values of $t_{0}$; under the alternative, both λ and $t_{0}$ could differ between clones. Note that the null did not fix λ at a single specific value; rather, it merely stipulated that λ, whatever value that may be, be shared by the clones being compared. We fitted the data to the null by finding the maximum likelihood estimates for each clone under the constraint of a shared value of λ and unconstrained values of $t_{0}$ and then summing their log likelihoods ($L_{Null}$). We fitted the alternative by imposing no constraints, and summing the two log-likelihoods ($L_{Alt}$). We defined $\Lambda_{Obs}=L_{Alt}-L_{Null}$. To obtain the distribution of Λ under the null, we simulated 500 datasets using the null parameter estimates, repeated the model-fitting exercise for each, and took the difference in the log-likelihood: $\Lambda_{Boot}$. The proportion of the bootstrap replicates where $\Lambda_{Boot}$ was greater than $\Lambda_{Null}$ was the approximate *P*-value for this comparison. To test for significant differences in $t_{0}$, we repeated this procedure, except $t_{0}$ was constrained and λ was allowed to vary.

To test whether the distribution of *P*-values calculated for each set of pairwise parameter comparisons diverged from the null expectation (*i.e.*, a uniform distribution), we binned the *P*-values into four categories [0.00,0.25), [0.25,0.50), [0.50,0.75), and [0.75,1.00] and conducted an exact multinomial test against expected frequencies in each bin under the null. The null bin frequencies were equal across the four categories (*i.e.*, 25% in each bin).

#### Burst size as a function of time:

We modeled the potential for burst size to change with time as a linear function, $D_{T}=\mu +\alpha T+\varepsilon$, where $D_{T}$ was the expected burst size when *T* minutes had elapsed since $t_{0}$, μ was the burst size at $t_{0}$, α was the rate of increase in burst size (*i.e.*, the slope parameter), and ε captured the normally distributed variation from expectation (mean 0, variance ${\text{σ}}^{2}$). Note that none of the terms in this model was observable. If a well sampled at time *t* showed a lysis event, we knew that lysis occurred before *t*, but we did not know exactly when. In addition, we did not know how many phage particles initially were in the well. Thus, both lysis time and burst size were hidden variables. In Appendix 3, we show that the observed count (rather than the burst size) could, like burst size, be expressed as a linear function:

$$C_{tar}=\alpha X_{tar}+\mu B_{tar}+\delta$$ (5)

where $C_{tar}$ was the count observed in well *r* sampled at time *t* from assay *a*, $B_{tar}$ was the number of phage particles in well *r* (assay *a*) that had lysed their hosts by time *t*, $X_{tar}$ was the sum of their lysis times, and δ was normally distributed with mean zero and variance $B_{tar}\sigma^{2}$. Our goal was to estimate the parameters μ, α, and σ, but because Equation 5 depended on the unobservable $X_{tar}$ and $B_{tar}$, we could not use standard regression. Instead, we employed an expectation maximization (EM) algorithm, through which the problem was circumvented by replacing $X_{tar}$ and $B_{tar}$ with their expected values before doing regression, and then iterating (Meng and Rubin 1993). More specifically, our EM algorithm involved the following steps: (1) guess values for $X_{tar}$ and $B_{tar}$; (2) estimate the parameters μ, α, and σ by least-squares regression (equivalent to maximum likelihood) assuming the guesses of $X_{tar}$ and $B_{tar}$ are correct; (3) assuming the parameter estimates are correct, calculate the expected values of $X_{tar}$ and $B_{tar}$, and update the guesses to these values; and (4) repeat steps 2 and 3 until convergence occurs. It turns out that both parameter estimation (step 2) and calculating expectations (step 3) involved a fair amount of math; we present this in Appendix 3.

To obtain confidence intervals and test for differences between clones in the regression parameters, we used a parametric bootstrap approach. For each genetically distinct clone, we simulated 1000 datasets using the estimated parameters and, each time, estimated α, μ and σ. We then sorted the bootstrap set of parameter values individually, using the 25th and the 975th values (*i.e.*, the central 95%) to define the confidence intervals. To keep our analysis simpler, we did not conduct formal likelihood ratio tests for significant differences between clones for these parameters. Instead, we compared confidence intervals and applied the conservative criteria that when confidence intervals were nonoverlapping, the two clones were significantly different.

Data and code required to repeat this analysis can be found in the Supplemental Material File S1.

### Data availability

Strains are available from D.M.W. upon request. File S1 contains a description of all supplemental material, including raw experimental data and the R code that was used to analyze them. Sequences of the WT (*Epos* mutant ancestor) and *D*-promoter ancestor are given by GenBank accession numbers J02482.1 and AF176034, respectively. Regions of the mutants' genomes were sequenced, and these sequences also were archived in GenBank (accession numbers KU646482 to KU646588).

## Results

Thirty-seven assays were conducted for the eight clones (four to six each). (Raw data from the assays are available in Supplemental Material, File S1.) The estimated Poisson parameter $\beta_{a}$ ranged from 0.20 to 0.91, with a mean of 0.46. It follows that approximately 63% of all wells had no phage particles, 29% had one particle, 6.5% had two particles, 1% had three, and 0.1% had four. Observed plaque counts ranged from zero to 1270, with eight being the smallest plaque count classified as representing a lysis event (see section *Statistical methods*).

Across the clones, the earliest times at which lysis was observed ranged from 11.0 min (*pos6*) to 14.5 min (*mut321*, *mut323*). Estimates of $t_{0}$ are shown in Table 3. This range of latent periods was consistent with past results, as reported in Bernhardt *et al.* 2002 (one-step growth assays in figure 2A in Bernhardt *et al.* 2002, about 12 min for wild type, *pos6*, and *pos4B* infections, though about 22min for *pos5*) and Brown *et al.* 2010 (about 12–14 min for the *D*-promoter mutants and wild type).

**Table 3:** Estimates of λ and $t_{0}$ for each clone

			Clone						
Clone	λ (min)	$t_{0}$ (min)	WT	319	321	323	324	5	6
WT	11.2 (7.5, 15.2)	12.2 (11.0, 13.2)							
319	5.8 (4.0, 7.8)	12.7 (12.0, 13.0)	0.028*						
321	4.8 (3.5, 6.2)	14.5 (14.0, 14.8)	0.002*	0.006*					
323	6.2 (4.5, 8.5)	13.9 (13.2, 14.2)	0.062**	0.132**	0.448***				
324	6.0 (4.2, 8.2)	13.4 (12.5, 13.5)	0.028*	0.548***	0.134**	0.698***			
5	5.7 (4.0, 7.5)	13.2 (12.5, 13.5)	0.010*	0.768***	0.082**	0.438***	0.778***		
6	8.9 (6.2, 12.2)	10.0 (9.0, 10.8)	0.012*	0.008*	< 0.002*	< 0.002*	< 0.002*	0.008*	
4B	9.5 (7.0, 12.5)	12.7 (11.8, 13.0)	0.800***	0.058**	0.010*	0.192***	0.140**	0.076**	< 0.002*

95% confidence intervals are in parentheses. The diagonal matrix to the right gives the *P*-values (based on 500 bootstrap replicates) of tests of the null hypothesis, which holds that the clones being compared have the same joint *λ* and $t_{0}$ values. * *P* < 0.05, ** *P* = 0.051–0.150, *** *P* > 0.151.

The smallest observed value of λ among the clones was 4.8 min (*mut321*), meaning that the cumulative probability of lysis after $t_{0}$ rose most rapidly for this clone under the experimental conditions. The wild type was found to have the largest λ (11.2 min). Values of λ for other clones, along with confidence intervals, can be found in Table 3.

**Figure 3:**
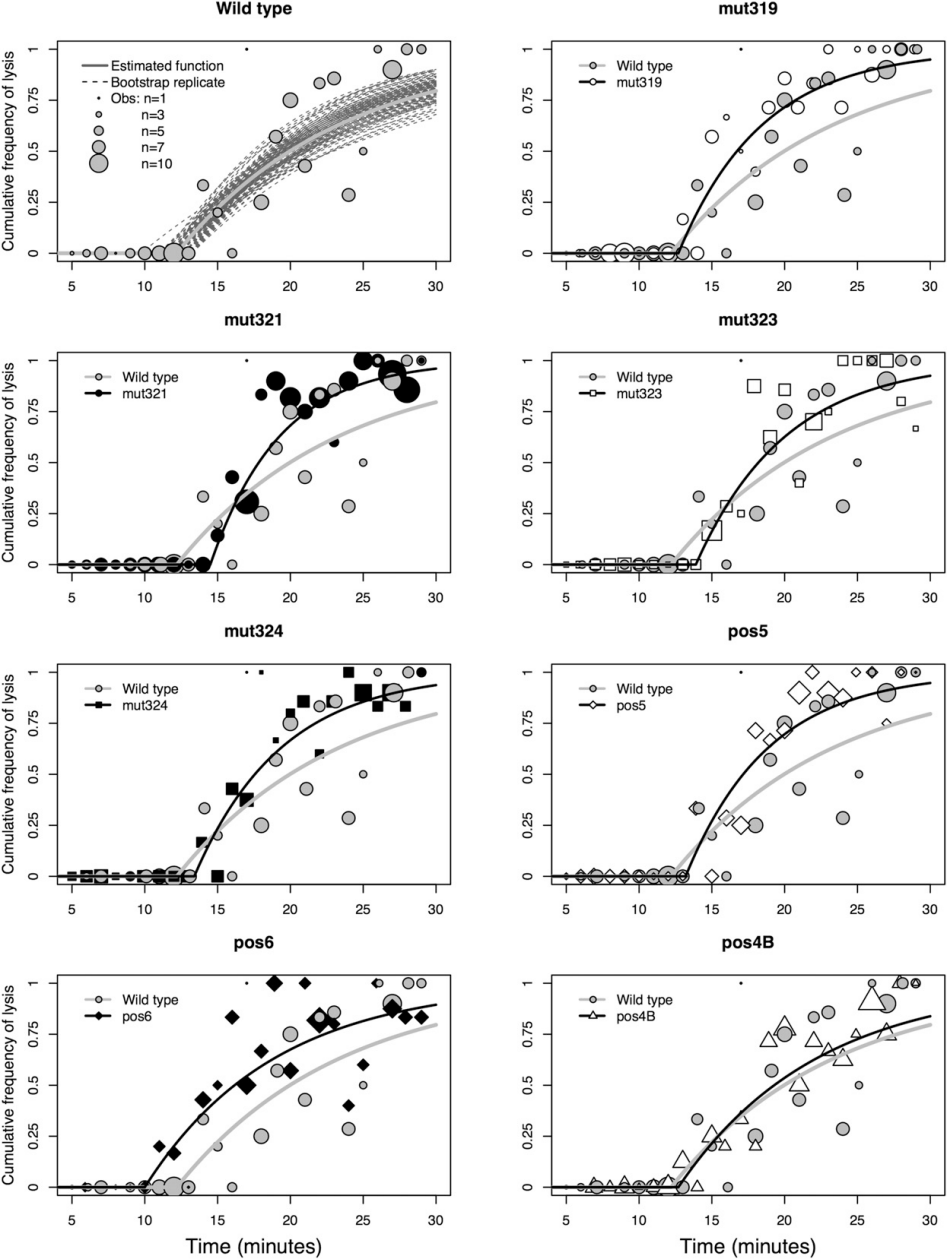
Estimated cumulative lysis probability by time functions, pairwise comparisons of each clone with the wild type. The wild type panel (top left) provides an example of fitting a cumulative lysis probability by time function to wild-type data. The gray line is the best-fit curve. Dotted lines represent 100 of the 500 bootstrap replicates. Each bubble’s position shows the proportion of wells sampled at a given time in which we observe a lysis event; the size of each bubble shows the number of samples on which the proportion is based. In all other panels, the wild type is represented with a gray line and gray bubbles.

**Figure 4:**
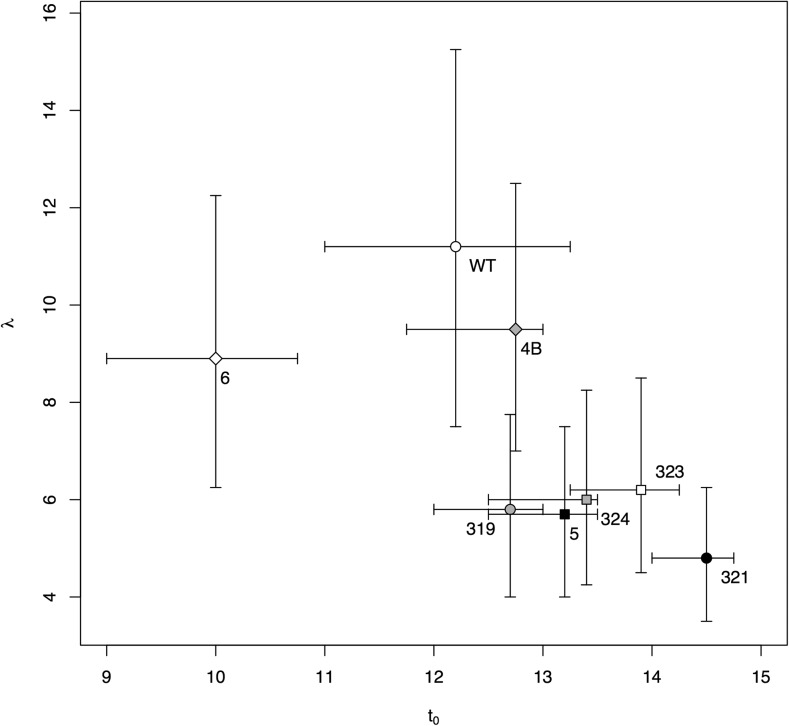
Estimates of parameters λ and $t_{0}$ that define the lysis probability function for wildtype and each mutant. Bars represent the approximate 95% confidence intervals for each parameter independent of the other (based on 500 bootstrap replicates).

Figure 3 illustrates how the observed increase over time in the proportion of wells showing lysis events was used to estimate the cumulative lysis probability function for each clone. The figure also includes pairwise comparisons of each clone with the wild type, demonstrating clear differences between them. Note that Figure 3 does not visualize the β parameter, which, in addition to the lysis events shown, was important in the estimation of lysis time phenotypes. Taken together, the curves form three rough clusters: (1) *pos6*, (2) wild type and *pos4B*, and (3) the remaining clones. This clustering can be observed clearly when the parameters composing those functions, λ and $t_{0}$, are plotted against each other (Figure 4).

These visual comparisons were formalized into pairwise statistical tests. Along with parameter estimates, Table 3 shows the pairwise *P*-values associated with the null hypothesis that the clones being compared came from the same function (*i.e.*, that jointly, both λ and $t_{0}$ are the same for the clones). If either parameter differed, this test would reject the null. By contrast, Table 4 shows the results of two different sets of tests: the set of pairwise tests of the null in which λ was held to be the same between clones, but $t_{0}$ was allowed to differ, and a similar test in which $t_{0}$ was held to be the same, but λ was allowed to differ. In other words, the first of these two sets of tests gauged differences between clones’ values of λ, whereas the second set of tests gauged differences between clones’ values of $t_{0}$.

**Table 4:** Approximate *P*-values of pairwise comparisons of individual parameters in lysis probability by time functions

	Clone							
Clone	WT	319	321	323	324	5	6	4B
WT		0.010*	< 0.002*	0.028*	0.018*	0.006*	0.322***	0.506***
319	0.478***		0.404***	0.888***	0.870***	0.844***	0.082**	0.028*
321	< 0.002*	0.006*		0.260***	0.314***	0.464***	0.010*	< 0.002*
323	0.050*	0.132**	0.246***		0.836***	0.702***	0.136**	0.092**
324	0.084**	0.334***	0.044*	0.486***		0.752***	0.078**	0.042*
5	0.210***	0.478***	0.014*	0.364***	0.698***		0.068**	0.022*
6	0.038*	0.002*	< 0.002*	0.002*	< 0.002*	0.004*		0.780***
4B	0.552***	0.910***	0.004*	0.142**	0.276***	0.478***	0.006*	

Above the diagonal are results of tests of the null in which *λ* is held to be the same between clones but $t_{0}$ is allowed to differ. Below the diagonal are results of similar tests in which $t_{0}$ is held to be the same but λ is allowed to differ. In other words, the first of these two sets of tests gauges differences between clones’ values of λ (above the diagonal), whereas the second set of tests gauges differences between clones’ values of $t_{0}$ (below the diagonal). Based on 500 bootstrap replicates each. * *P* < 0.05, ** *P* = 0.051–0.150, *** *P* > 0.151.

Notice that we have not corrected for multiple tests. Our *P*-values are based on 500–1000 bootstrap replicates, whereas the amount of replication needed to achieve significance under a Bonferroni correction would be in the 5000–10,000 range and computationally infeasible. We note, however, that if the joint null hypothesis were universally true (*i.e.*, all clones followed the same function), we would expect the *P*-values of the 28 pairwise tests to follow a uniform distribution, and we would expect just one or two false positives (28 × 0.05 = 1.4). Indeed, we would be unlikely to have more than three false positives, since the binomial probability of greater than three false rejections is 0.0491. Instead, the distribution of *P*-values for the joint λ-$t_{0}$ comparisons skewed strongly from a uniform distribution [exact multinomial test, *P* < 0.0001 (McDonald 2014)], and we observed 13 pairwise results less than or equal to 0.05 (Table 3). [The binomial probability of 13 or more false rejections is $\sum_{i=13}^{28}\left(\begin{array}{c} 28\\ i \end{array}\right)0.05^{i}{\left(1-0.05\right)}^{28-i}=2.24\times 10^{-10}$.] We suggest, therefore, that most, though perhaps not all, of the differences that appeared as significant were truly significant. Similarly, the tests for pairwise differences in only λ and only $t_{0}$ yielded 10 and 13 values less than or equal to 0.05, respectively, which is far more than expected under the null of a uniform distribution (exact multinomial test, *P* = 0.0068 and *P* < 0.0001, respectively). (The probability of 10 or more false rejections is $5.56\times 10^{-7}$, and the probability of 13 or more is $2.24\times 10^{-10}$; Table 4.)

We also note that our omission of adsorption time from our statistical model (see *Materials and Methods*) had little effect on these findings. By ignoring adsorption time, we treated the time to lysis as a single exponential process, when in reality it involves the sum of two exponential waiting times (Abedon *et al.* 2001). However, additional analysis (not shown) demonstrated that, because adsorption happens at least an order of magnitude faster than lysis (Brown *et al.* 2013), the distribution of lysis time under the one-exponential model is very similar to that generated by a more complex two-exponential model. Furthermore, the variance of our one-exponential model is slightly larger than that of the two-exponential model, which implies that tests underlying the pairwise phenotypic comparisons were conservative. To see why, recall that the variance of an exponential with mean λ is $\lambda^{2}$. The variance of the sum of two independent exponentials with means $\lambda_{lysis}$ and ${\text{λ}}_{\text{adsorb}}$ is the sum of their individual variances: $\lambda_{lysis}^{2}+\lambda_{adsorb}^{2}$. But the variance of a single exponential with mean $\lambda =\lambda_{lysis}+\lambda_{adsorb}$ is ${\left(\lambda_{lysis}+\lambda_{adsorb}\right)}^{2}=\lambda_{lysis}^{2}+\lambda_{adsorb}^{2}+2\lambda_{lysis}\lambda_{adsorb}$. Therefore, although we might have gained marginally more power in our phenotypic estimates by adopting a more complex two-exponential statistical model (supplemented with data on adsorption) or following the preattachment protocols described in Brown *et al.* 2013 and Cherwa Jr *et al.* 2009, the methods we employed were adequate.

**Figure 5:**
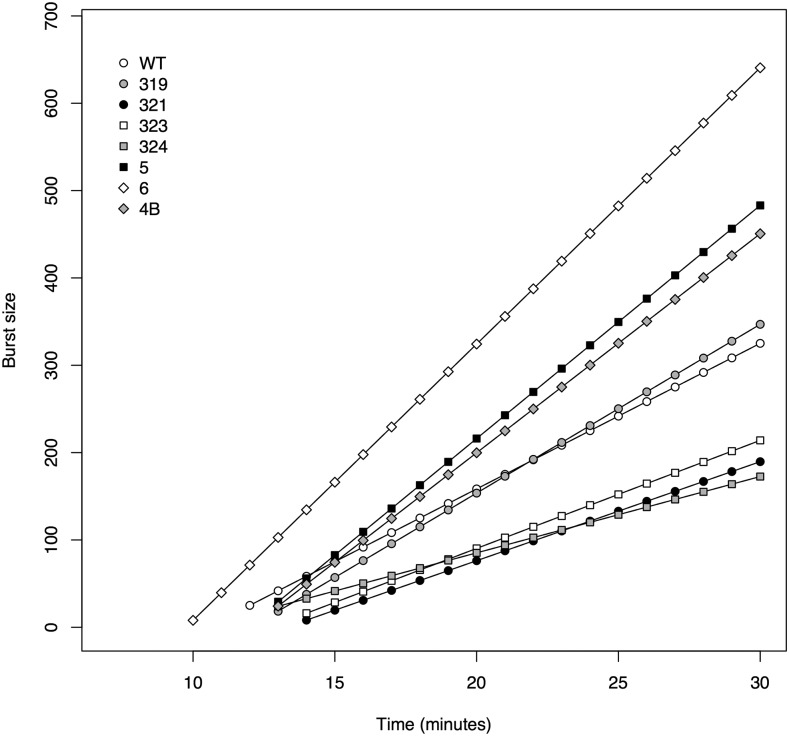
Estimated burst size by time relationships for wild type and each mutant.

The regression analysis of burst size by time revealed that burst size increased with time for all clones (Figure 5). The parameter estimates for each clone are provided in Table 5 along with confidence intervals. Figure 5 illustrates these regression parameter estimates graphically, showing that clones fell into four clusters of decreasing slope: (1) *pos6*, (2) *pos4B* and *pos5*, (3) wild type and *mut319*, and (4) *mut321*, *mut323*, and *mut324*. Formal testing of parameter differences was not conducted. However, where confidence intervals were nonoverlapping, there was good evidence for a significant difference. For slope, the confidence intervals within the four clusters were generally overlapping while intervals between clusters were not.

**Table 5:** Parameter estimates and confidence intervals for burst size by time regression analysis

Clone	Slope ($\hat{\alpha }$) (PFU/Min)	Intercept ($\hat{\mu }$) (PFU)	$\hat{\sigma }$ (PFU)
WT	16.7 (15.1, 18.4)	28.4 (19.9, 36.5)	19.9 (15.6, 23.5)
319	19.3 (19.0, 19.6)	12.6 (11.4, 13.8)	3.2 (2.6, 3.7)
321	11.3 (10.7, 12.0)	13.9 (11.6, 16.1)	7.4 (6.2, 8.3)
323	12.4 (11.7, 13.1)	14.8 (12.1, 17.5)	7.2 (5.8, 8.3)
324	8.7 (7.6, 9.8)	27.5 (23.0, 32.3)	13.0 (10.7, 15.2)
5	26.7 (25.0, 28.3)	34.6 (28.1, 41.0)	17.5 (14.4, 20.2)
6	31.6 (31.1, 32.2)	8.0 (5.3, 10.7)	7.2 (5.8, 8.4)
4B	25.1 (24.5, 25.6)	16.7 (13.9, 19.2)	6.6 (5.3, 7.7)

Each confidence interval based on 1000 bootstrap replicates.

Table 6 summarizes the calculated mean and variance in lysis time for each clone.

**Table 6:** Estimates of lysis time mean and variance of **φ**X174 clones

Clone	Lysis Time	
	Mean (min)	Variance (min^2^)
WT	23.4 (18.5, 28.4)	125.4 (56.3, 231.0)
319	18.5 (16.0, 20.8)	33.6 (16.0, 60.8)
321	19.3 (17.5, 21.0)	23.0 (12.3, 38.4)
323	20.1 (17.7, 22.7)	38.4 (20.3, 72.3)
324	19.4 (16.7, 21.7)	36.0 (17.6, 67.2)
5	18.9 (16.5, 21.0)	32.5 (16.0, 56.3)
6	18.9 (15.2, 23.0)	79.2 (38.4, 148.8)
4B	22.2 (18.8, 25.5)	90.3 (49.0, 156.3)

95% confidence intervals are in parentheses. Mean lysis time = $t_{0}+\text{λ}$. Variance in lysis time = ${\text{λ}}^{2}$.

## Discussion

In recent years, it has been found that the probability that a beneficial mutation will fix in a lytic phage population depends sensitively on the life history parameter it affects (Wahl and DeHaan 2004; Hubbarde *et al.* 2007; Patwa and Wahl 2008, 2009). Furthermore, analytical work has demonstrated that variance in life history traits, not just the trait means, can influence growth rate predictions, particularly in iteroparous populations far from a stable age distribution (Bull *et al.* 2011). Inspired by this body of work, we wished to learn more about whether bacteriophage life history traits, including higher moments (*e.g.*, lysis time variance), could be subject to the phage’s genetic control.

To this end, we used a serially sampled parallel infection assay to interrogate the lysis time and burst size phenotypes of a panel of eight genetically distinct φX174 clones. Among these clones were four with mutations in the *D* promoter that have been reported to downregulate the transcription of several genes, including the lysis gene (*E*); three *Epos* clones with mutations in *E* that have been found to upregulate its expression; and the wild-type ancestor of the *Epos* mutants. (As noted in the *Materials and Methods*, these mutations were introduced on slightly different genetic backgrounds.)

Using a novel statistical approach that allowed us to overcome the challenge of hidden variables, we found significant variation in both lysis time and burst size among these clones, including evidence that φX174 exerts genetic control over lysis time variance under our experimental conditions.

The inferred differences among the clones’ burst size phenotypes are generally consistent with our limited understanding of the mutations’ biological effects (Table 5 and Figure 5). In accordance with their depressed rates of transcription at the *D* promoter (Brown *et al.* 2010), burst size increased more slowly than the wild type in three of the four *D*-promoter mutants. (Burst size increased with lysis time at about the same rate as the wild type in the fourth *D*-promoter mutant, *mut319*.) By contrast, burst size increased more quickly than the wild type in the three *Epos* mutants. This may be related to the findings of Bernhardt *et al.* 2002 that the R3H and L19F mutations lead to markedly faster E protein synthesis than the wild type, perhaps by increasing mRNA translatability. Given the compact genome of φX174 and the location of *E* within the *D* gene (Figure 2), it is possible that these mutations somehow upregulate gene expression more globally, thereby precipitating more rapid accumulation of progeny virions and larger burst sizes.

As with burst size, we inferred significant variation in lysis time phenotypes among the eight clones under the tested experimental conditions. The clones seem to differ in their overall cumulative lysis probability functions (Table 3) and in both $t_{0}$ and λ individually (Table 4).

Within these differences, we observe an inverse relationship between $t_{0}$ and λ (Figure 4): under the tested conditions, the mutations appear to delay the onset of lysis while causing the cumulative lysis probability to rise more quickly compared to the wild type (Figure 3). (The most noteworthy exception is *pos6*, the only clone with a value of $t_{0}$ less than that of the wild type.) A consequence of this trend is that, despite substantial differences in $t_{0}$ and λ, the clones’ mean lysis times (computed as the sum of $t_{0}$ and λ) are rather similar (Table 6). A mechanistic investigation of the apparent maintenance of mean lysis time is beyond the scope of this study, but we speculate that it may reflect constraints on lysis timing imposed by the host’s cell cycle (for φX174: Witte *et al.* 1998; Young *et al.* 2000; and other phages as well: Hadas *et al.* 1997; Storms *et al.* 2014). While mutations in *E* and related genes could potentially bias lysis timing earlier or later, or make the process more or less noisy, the host’s physiology and cell cycle may prevent extreme divergence from a common mean.

In fact, given the genetic and phenotypic constraints on φX174 life history, it is notable that burst size and lysis time phenotypes appear to diverge as much as they do. A primary genetic constraint is the phage’s compact genomic architecture. The *E* gene is entirely embedded within the structurally essential *D* gene (Barrell *et al.* 1976; Figure 2), and the *D* promoter simultaneously regulates the transcription of *E*, *D*, and four other structural genes downstream (*i.e.*, *J*, *F*, *G*, and *H*) (Fane *et al.* 2006). The work presented here stands with and expands upon previous studies (*e.g.*, Brown *et al.* 2010, 2013; Bernhardt *et al.* 2002) in demonstrating that, despite the relatively limited genotype space accessible to the organism, the genomic architecture of φX174 permits a significant degree of flexibility in lysis time and burst size phenotypes. Still, the genetic constraints on these phenotypes are clearly visible in the form of pleiotropic effects on burst size and lysis time in our data.

The inferred disparity among our clones’ lysis time parameters is also interesting given the aforementioned phenotypic constraints imposed by the host’s cell cycle and physiology. Whereas λ and many other larger DNA phages effect lysis via a tightly regulated, two-protein lysis strategy, φX174 and other *Microviridae* lyse their hosts by way of a single protein (Young *et al.* 2000; Young and Wang 2006). The lysis timing of φX174 is thought to be dictated largely by the timing of host cell division and, therefore, compared with phages like λ, even less subject to the phage’s genetic control (Witte *et al.* 1998; Young *et al.* 2000; Zheng *et al.* 2008; Dennehy and Wang 2011; Singh and Dennehy 2014; Storms *et al.* 2014). But our demonstration of genetic control of both lysis time mean and variance hints at the possibility of as-yet-unknown mechanisms exerting some control over phage lysis timing. Indeed, researchers have long speculated that protein E may mediate lysis timing not just through its interactions with MraY, but also through interactions with the host protein SlyD (Young and Wang 2006). SlyD plays an important role in cell division and cell cycle regulation (Roof *et al.* 1997) and is required for stability of wild-type E protein (Bernhardt *et al.* 2002).

### Strengths and limitations of our experimental and statistical approach

The strength of the experimental and statistical approach taken in this study lies in its power and applicability. It is powerful in that, more effectively than most other methods, it simultaneously captures information related to lysis time variance and burst size as a function of time. The approach also has potential application to a range of microbiological questions. The principles and tools underlying the assay and inferential apparatus could be applied usefully to other cases in which one seeks to make inferences about dynamic, Poisson-distributed processes that are sampled destructively over a time course (*e.g.*, tracking the fates of individual tagged mutants in a dynamic population of cells or viruses).

However, we concede our approach has limitations. There is increasing evidence that host cells’ physiology plays a vital role in determining phage infection dynamics (Hadas *et al.* 1997; Rabinovitch *et al.* 2002; Storms *et al.* 2014; Bull *et al.* 2014). Yet, in synchronizing and rapidly sampling infections, our assay exposed the cells to temperature changes and nutritional regimes on a short timescale, which may have altered cells’ physiology in ways important for the measured phenotypes (van Elsas *et al.* 2011). While these sorts of manipulations are not uncommon in the literature (*e.g.*, Wang 2006; Cherwa Jr *et al.* 2009), they limit the generality of some conclusions drawn from this work. The unique conditions of the assay complicate comparisons to previous estimates of the clones’ burst size and lysis time phenotypes, and they make it impractical to validate our methods against traditional single-step growth experiments with much confidence.

By the same token, the lysis times and burst sizes inferred in this work may differ from those φX174 exhibits in nature. Environmental differences in factors like host density and diversity, as well as temperature changes and nutritional regimes, could possibly lead to differences between phenotypes measured in the assay and in the wild. That said, it is unclear how well any particular assay approximates the phage’s natural environment, which remains largely uncharacterized (Michel *et al.* 2010). Further study of the ecological niche of φX174 would be necessary to clarify which environmental differences between our assay and the wild are most relevant to the phage’s life history phenotypes.

Finally, our assumption of a linear increase in burst size with time, though substantiated by significant past work with φX174 and other phages (Hutchison and Sinsheimer 1966; Wang *et al.* 1996; Wang 2006), may be subject to debate (Storms *et al.* 2014). We hope further research will help better evaluate the appropriateness of this assumption.

When it comes to achieving the core aims of this work, we believe the strengths of our experimental and statistical approach outweigh its limitations. It allowed us to demonstrate that, under at least one set of conditions held constant for eight similar but distinct φX174 clones, burst size and lysis time phenotypes (including lysis time variance) differed significantly. Furthermore, it is reasonable to assume that the degree of genetic variability among the clones is comparable to that which is the substrate for natural selection acting in the wild.

### Evolutionary implications of heritable variation in lysis time variance

Our finding that variance in the lysis time of φX174 can be subject to at least some genetic control inspired us to ask, “What, then, could be the evolutionary implications of mutations affecting variance in phage lysis time?” Trivially, if burst size grows more slowly than exponentially once a cell has been infected, then a mutation increasing lysis time variance will be selectively favored, because early lysis events contribute more to population growth than late lysis events detract (Bull *et al.* 2011; but see Singh and Dennehy 2014). Empirically, phage burst size is thought to increase linearly (Hutchison and Sinsheimer 1966; Wang *et al.* 1996; Wang 2006; but see Storms *et al.* 2014), immediately suggesting one mode of selection. Mutations increasing lysis time variance in a given environment likely would fix quickly under this mode of selection.

In order to control for the influence of this mode of selection, we recently have explored a model in which burst size increases exponentially in time since infection (D. M. Weinreich, C. Brown, and L. M. Wahl, unpublished data). This has allowed us to infer the intrinsic selective consequences of mutations that influence lysis time variance. As expected, in phage populations growing at their stable age-of-infection distribution in unlimited host cells (Bull *et al.* 2011; Bull 2006), we found such mutations to be selectively neutral, since, by construction, the contribution of early bursts to population growth is now exactly balanced by (exponentially larger) late bursts.

However, the moment a mutation appears, the mutant subpopulation is by definition not at stable age distribution, and indeed we have found in simulations that mutations increasing variance in lysis time are transiently enriched in the population (D. M. Weinreich and C. Brown, unpublished data). This effect follows from the fact that the frequency of phages with high lysis time variance exceeds (sometimes considerably) the frequency of their low-variance brethren early in each lysis cycle. While the reverse is true late in the cycle, the effect is of smaller magnitude and much shorter lived. Finally, overlaying periodic demographic bottlenecks (*e.g.*, as employed in lab conditions, but also surely in nature), we observed that mutations increasing lysis time variance can enjoy a dramatic increase in population frequency, despite their neutrality when they finally reach their stable age-of-infection distribution (D. M. Weinreich and C. Brown, unpublished). We therefore suggest a second mode of selection acting on mutations affecting variance in lysis time.

### Future directions

The findings reported here suggest two complementary avenues for future investigation. One is microbiological, and it involves exploring φX174 lysis in relation to its genetic determinants and its host’s biology. It would be interesting to explore the mutants’ performance in a range of assays, such as those involving thermally regulated microscopy (Dennehy and Wang 2011), single-cell microfluidics-based techniques (Yin and Marshall 2012), or periodic filtration and sampling (Hutchison and Sinsheimer 1963). (The last and oldest of these techniques could prove especially fruitful, since it would allow direct access to both lysis time and burst size.) In addition, it would be highly informative to use methods like those designed by Storms *et al.* 2014 to study how φX174 mutants’ burst sizes and lysis times vary as a direct function of the host’s cell cycle. These types of experiments could yield valuable insight into the regulation and host-dependence of φX174 life history phenotypes.

Theoretical evolutionary problems present another opportunity for future research. Does natural selection act on lysis time variance in nature? What is the fixation probability of a mutation that affects lysis time variance, and how do populations carrying such mutations evolve in the face of periodic bottlenecks? Existing computational models could be strengthened by more thoroughly exploring the models’ parameter sensitivity and investigating how mutations that pleiotropically influence adsorption, lysis time, and burst size affect predicted population dynamics. In addition, the models must be integrated with other theoretical work exploring the fixation probabilities of mechanistically distinct beneficial mutations in lytic phages (Wahl and DeHaan 2004; Hubbarde *et al.* 2007; Patwa and Wahl 2008, 2009). Once elaborated and refined, models could be tested empirically through both “single-sampling” assays and longer-term evolution experiments. The former approach would assess the probability of loss of lysis time variants introduced at very low frequency in replicate phage populations, while the latter would explore whether real evolutionary outcomes match our theoretical predictions.

## Supplementary Material

Supporting Information

## Appendix 1: Number of Phages per Well Poisson Estimator

The counts from an assay place each well into one of three categories: empty, not showing lysis events but nonzero, and showing lysis events. The probability of a well being empty is $e^{-\beta_{a}}$. The probability that a well has *n* phages is $\beta_{a}^{n}e^{-\beta_{a}}/n!$. The probability that all *n* phages fail to lyse their host is $p^{n}$, leading the probability of *n* in a well showing no lysis events to be ${\left(p\beta_{a}\right)}^{n}e^{-\beta_{a}}/n!$. Finally, the probability of a well showing one or more lysis events is one minus the probability of a well being empty, plus the sum of all the probabilities associated with wells that do not show lysis events, which turns out to be $1-e^{-\left(1-p\right)\beta_{a}}$. The categorization of wells into three types leads to the following likelihood.

$$\begin{array}{c} L\left(N_{a1},N_{a2},\cdots ,N_{aM_{a}},R_{a}\right)=\frac{\prod_{r=1}^{M_{a}}{\left(p\beta_{a}\right)}^{N_{ar}}e^{-\beta_{a}}}{N_{ar}!}\cdot \left(\begin{array}{c} L_{a}\\ R_{a} \end{array}\right){\left(1-e^{-\left(1-p\right)\beta_{a}}\right)}^{R_{a}}\\ =\frac{{\left(p\beta_{a}\right)}^{\sum N_{ar}}e^{-M_{a}\beta_{a}}}{\prod_{r=1}^{M_{a}}N_{ar}!}\cdot \left(\begin{array}{c} L_{d}\\ R_{a} \end{array}\right){\left(1-e^{-\left(1-p\right)\beta_{a}}\right)}^{R_{a}} \end{array}$$ (6)

Therefore,

$$\ln L=\sum_{r=1}^{M_{a}}N_{ar}\left(\ln\beta_{a}+\ln p\right)-M_{a}\beta_{a}-\sum_{r=1}^{M_{a}}\ln N_{ar}!\;+\ln\left(\begin{array}{c} L_{a}\\ R_{a} \end{array}\right)+R_{a}\ln\left(1-e^{-\left(1-p\right)\beta_{a}}\right)$$ (7)

The maximum likelihood estimator is obtained by first calculating two partial derivatives,

$$\frac{\partial }{\partial \beta_{a}}\ln\left(L\right)=\frac{\sum_{r=1}^{M_{a}}N_{ar}}{\beta_{a}}-M_{a}+\frac{R_{a}\left(1-p\right)e^{-\left(1-p\right)\beta_{a}}}{1-e^{-\left(1-p\right)\beta_{a}}}$$ (8)

$$\frac{\partial }{\partial p}\ln\left(L\right)=\frac{\sum_{r=1}^{M_{a}}N_{ar}}{p}-\frac{R_{a}\beta_{a}e^{-\left(1-p\right)\beta_{a}}}{1-e^{-\left(1-p\right)\beta_{a}}}$$ (9)

and then setting both $\frac{\partial }{\partial \beta_{a}}\ln\left(L\right)=0$ and $\frac{\partial }{\partial p}\ln\left(L\right)=0$. Denote the solution by ${\hat{\beta }}_{a}$ and $\hat{p}$, respectively. The solution associated with Equation 8 leads to

$$\hat{\beta_{a}}=\bar{N_{a}}+\frac{R_{a}}{M_{a}}\frac{\left(1-\hat{p}\right)\hat{\beta_{a}}e^{-\left(1-\hat{p}\right)\hat{\beta_{a}}}}{1-e^{-\left(1-\hat{p}\right)\hat{\beta_{a}}}}$$ (10)

and the solution associated with Equation 9 gives

$$\frac{\bar{N_{a}}}{\hat{p}}=\frac{R_{a}}{M_{a}}\frac{\hat{\beta_{a}}e^{-\left(1-\hat{p}\right)\hat{\beta_{a}}}}{1-e^{-\left(1-\hat{p}\right)\hat{\beta_{a}}}}.$$ (11)

Now multiply both sides of the above equation by $1-\hat{p}$

$$\frac{\bar{N_{a}}\left(1-\hat{p}\right)}{\hat{p}}=\frac{R_{a}}{M_{a}}\frac{{\hat{\beta }}_{d}\left(1-\hat{p}\right)e^{-\left(1-\hat{p}\right)\hat{\beta_{a}}}}{1-e^{-\left(1-\hat{p}\right)\hat{\beta_{a}}}}$$ (12)

Note that second term on the right side of Equation 10 is equivalent to the left side of Equation 12. This leads to the following simple relationship between ${\hat{\beta }}_{a}$ and $\hat{p}$:

$$\hat{\beta_{a}}={\bar{N}}_{a}+{\bar{N}}_{a}\frac{1-\hat{p}}{\hat{p}}$$ (13)

Thus $\hat{p}={\bar{N}}_{a}/{\hat{\beta }}_{a}$ and $\left(1-\hat{p}\right){\hat{\beta }}_{a}={\hat{\beta }}_{a}-{\bar{N}}_{a}$. We can eliminate $\hat{p}$ from Equation 10 by substituting ${\hat{\beta }}_{a}-{\bar{N}}_{a}$ for $\left(1-\hat{p}\right){\hat{\beta }}_{a}$ to get

$$\begin{array}{c} \hat{\beta_{a}}=\bar{N_{a}}+\frac{R_{a}}{M_{a}}\frac{\left({\hat{\beta }}_{a}-{\bar{N}}_{a}\right)e^{-\left({\hat{\beta }}_{a}-{\bar{N}}_{a}\right)}}{1-e^{-\left({\hat{\beta }}_{a}-{\bar{N}}_{a}\right)}}\\ \hat{\beta_{a}}-\bar{N_{a}}=\frac{R_{a}}{M_{a}}\frac{\left({\hat{\beta }}_{a}-{\bar{N}}_{a}\right)e^{-\left({\hat{\beta }}_{a}-{\bar{N}}_{a}\right)}}{1-e^{-\left({\hat{\beta }}_{a}-{\bar{N}}_{a}\right)}}\\ \frac{M_{a}}{R_{a}}=\frac{e^{-\left({\hat{\beta }}_{a}-{\bar{N}}_{a}\right)}}{1-e^{-\left({\hat{\beta }}_{a}-{\bar{N}}_{a}\right)}}\\ \frac{M_{a}/R_{a}}{1+M_{a}/R_{a}}=e^{-\left({\hat{\beta }}_{a}-{\bar{N}}_{a}\right)} \end{array}$$ (14)

Solving Equation 14 for ${\hat{\beta }}_{a}$ gives the estimator,

$${\hat{\beta }}_{a}={\bar{N}}_{a}+\ln\left(M_{a}+R_{a}\right)-\ln\left(M_{a}\right)$$ (15)

## Appendix 2: Time to Lysis

Recall that we model the probability of a phage lysing its host by time *t* with a shifted exponential, $P\left(T<t\right)=w_{t}=1-e^{-\left(t-t_{0}\right)/\lambda }$. Define $B_{tar}$ as the number of phages in well *r*, assay *a*, that have lysed their hosts by time *t* (when sampling occurs). $B_{tar}$ is Poisson with mean $\beta_{d}w_{t}$. Let $Y_{tar}$ be an indicator with value 1 if $B_{tar}>0$ and 0 otherwise. Let $U_{tar}$ be the number of phages that have not lysed their hosts in well *r*, assay *a*, at time *t* ($U_{tar}=N_{ar}-B_{tar}$). Based on standard theory $U_{tar}$ is independent of $B_{tar}$ and $U_{tar}$ is also Poisson distributed but with mean $\beta_{a}\left(1-w_{t}\right)$. However, $U_{tar}$ is only directly observable when no phages in the well have lysed their hosts (*i.e.*, when $B_{\text{tar}}=0$). We now show how to use the binary $Y_{\text{tar}}$ together with Poisson distributed $U_{\text{tar}}$ to estimate λ and $t_{0}$. First, the likelihood of the lysis data are given by,

$$L\left(Y\right)=\prod_{a}\prod_{t}\prod_{r}{\left(e^{-\beta_{a}w_{t}}\right)}^{1-Y_{tar}}{\left(1-e^{-\beta_{a}w_{t}}\right)}^{Y_{tar}}$$ (16)

The likelihood for the number of phages in wells not showing lysis events is given by

$$L\left(U|Y=0\right)=\prod_{a}\prod_{t}\prod_{r}\frac{{\left(\left(1-w_{t}\right)\beta_{a}\right)}^{U_{tar}}}{U_{tar}!}e^{-\beta_{d}\left(1-w_{t}\right)}$$ (17)

However, to account for the fact that $U_{\text{tar}}$ is only observable when no lysis occurs (*i.e.*, $Y_{\text{tar}}=0$), we must adjust the likelihood for wells showing plaques but not showing lysis events as follows.

$$L\left(U|Y\right)=\prod_{a}\prod_{t}\prod_{r}\frac{{\left(\left(1-w_{t}\right)\beta_{a}\right)}^{U_{tar}\left(1-Y_{tar}\right)}}{\left(\left(1-Y_{tar}\right)U_{tar}\right)!}e^{-\beta_{d}\left(1-w_{t}\right)\left(1-Y_{tar}\right)}$$ (18)

The combined likelihood is,

$$\begin{array}{c} L\left(Y,U\right)=\prod_{a}\prod_{t}\prod_{r}{\left(e^{-\beta_{a}w_{t}}\right)}^{1-Y_{tar}}\\ {\left(1-e^{-\beta_{a}w_{t}}\right)}^{Y_{tar}}\frac{{\left(\left(1-w_{t}\right)\beta_{a}\right)}^{U_{tar}\left(1-Y_{tar}\right)}}{\left(\left(1-Y_{tar}\right)U_{tar}\right)!}e^{-\beta_{a}\left(1-w_{t}\right)\left(1-Y_{tar}\right)} \end{array}$$ (19)

Dropping factorial terms that do not involve parameters and taking the log, the log-likelihood is proportional to

$$\begin{array}{c} \ln L\left(Y,U\right)∼\sum_{a}\sum_{t}\sum_{r}-\left(1-Y_{tar}\right)\beta_{a}w_{t}+Y_{tar}\ln\left(1-e^{-\beta_{a}w_{t}}\right)\\ +\left(1-Y_{tar}\right)\left(U_{tar}\ln\left(\left(1-w_{t}\right)\beta_{a}\right)-\beta_{a}\left(1-w_{t}\right)\right) \end{array}$$ (20)

As discussed in the main text, we estimate λ and $t_{0}$ by brute force, where we calculate the likelihood using Equations 3 and 20 for a large array of joint values, taking the largest as the (approximate) maximum likelihood estimates.

## Appendix 3: Burst Size as a Function of Size

The count observable in well *r* from assay *a* taken at time *t*, $C_{\text{tar}}$, can be expressed as,

$$C_{tar}=\alpha \sum_{i=1}^{N_{d\cdot }}T_{iar}I\left\{T_{tar}<t\right\}+\mu \sum_{i=1}^{N_{d\cdot }}I\left\{T_{iar}<t\right\}+\sum_{i=1}^{N_{d\cdot }}\int_{i}I\left\{T_{iar}<t\right\}$$ (21)

where $T_{\text{iar}}$ is the lysis time of phage *i* in well *r* of assay *a*, and *I* represents an indicator function that takes value 1 when the condition that follows is true, and 0 otherwise. This simplifies to, $C_{tar}=\alpha X_{tar}+\mu B_{tar}+\delta$ where $B_{\text{tar}}$ is the number of phages initially in well *r* of assay *a* sampled at time *t*, $X_{tar}$ is the sum of their lysis times, and δ is normally distributed with mean zero and variance $B_{\text{tar}}{\text{σ}}^{2}$. Our goal is to then estimate μ, α, and σ. Because $X_{\text{tar}}$ and $B_{\text{tar}}$ are unobservable but are needed to estimate the parameters, we employ the following EM algorithm which replaces them with their expected value. Note that these steps summarize the process; we provide derivations and details in subsections below.

### EM algorithm

1. Begin with initial guesses for $B_{tar}^{\left(0\right)}$ and $X_{tar}^{\left(0\right)}$ for each well. Let j=0.
2. Given these values, estimate α and μ using least-squares.
  $$\underset{\alpha ,\mu }{\min}\sum_{t,a,r}\frac{{\left(C_{tar}-\alpha X_{tar}^{\left(j\right)}-\mu B_{tar}^{\left(j\right)}\right)}^{2}}{B_{tar}^{\left(j\right)}}=\sum_{t,d,r}\frac{{\left(C_{tar}-\hat{\alpha_{j}}X_{tar}^{\left(j\right)}-\hat{\mu_{j}}B_{tar}^{\left(j\right)}\right)}^{2}}{B_{tar}^{\left(j\right)}}$$ (22)
3. Use the least-squares estimates $\hat{{\text{α}}_{j}}$ and $\hat{{\text{μ}}_{j}}$ to estimate ${\text{σ}}^{2}$
  $${\hat{\sigma }}_{j}^{2}=\frac{1}{n}\sum_{t,a,r}\frac{{\left(C_{tar}-\hat{\alpha_{j}}X_{tar}^{\left(j\right)}-\hat{\mu_{j}}B_{tar}^{\left(j\right)}\right)}^{2}}{B_{tar}^{\left(j\right)}}$$ (23)
4. Impute the values $X_{tar}^{\left(j\right)}$ and $B_{tar}^{\left(j\right)}$ using their posterior expected values. Details below.
  $$X_{tar}^{\left(j+1\right)}=E\left(X_{tar}^{\left(j\right)}|C_{tar},\hat{\alpha_{j}},\hat{\mu_{j}},{\hat{\sigma }}^{2}\right)$$ (24)
  $$B_{tar}^{\left(j+1\right)}=E\left(B_{tar}^{\left(j\right)}|C_{tar},\hat{\alpha_{j}},\hat{\mu_{j}},{\hat{\sigma }}^{2}\right)$$ (25)
5. Return to step 2, replacing *j* with j+1, until convergence occurs.

### Least squares estimates for α and μ (step 2 in EM algorithm)

Because the error is normal, values of μ and α that minimize the sum of squared differences between observed and expected counts provide the maximum likelihood estimates. The function that expresses this sum of squares is,

$$g\left(\alpha ,\mu \right)=\sum_{t,a,r}\frac{{\left(C_{tar}-\alpha X_{tar}-\mu B_{tar}\right)}^{2}}{B_{tar}}$$ (26)

Then

$$\begin{array}{l} \frac{\partial }{\partial \alpha }g\left(\alpha ,\mu \right)=\sum_{t,a,r}\frac{-2X_{tar}}{B_{tar}}\left(C_{tar}-\alpha X_{tar}-\mu B_{tar}\right)\\ \frac{\partial }{\partial \mu }g\left(\alpha ,\mu \right)=\sum_{t,a,r}-2\left(C_{tar}-\alpha X_{tar}-\mu B_{tar}\right) \end{array}$$ (27)

Set $\frac{\partial }{\partial \alpha }g\left(\alpha ,\mu \right)=0$ and $\frac{\partial }{\partial \mu }g\left(\alpha ,\mu \right)=0$ solve. The solutions are the least squares estimates denoted by $\hat{\alpha }$ and $\hat{\mu }$ satisfy the following set of linear equations.

$$\begin{array}{c} \sum_{t,a,r}\frac{X_{tar}C_{tar}}{B_{tar}}=\hat{\alpha }\sum_{t,a,r}\frac{X_{tar}^{2}}{B_{tar}}+\hat{\mu }\sum_{t,a,r}X_{tar}\\ \sum_{t,a,r}C_{tar}=\hat{\alpha }\sum_{t,a,r}X_{tar}+\hat{\mu }\sum_{t,a,r}B_{tar} \end{array}$$ (28)

For notational convenience let

$$\begin{array}{l} A=\sum_{t,a,r}\frac{X_{tar}^{2}}{B_{tar}}\;\;B=\sum_{t,a,r}X_{tar}\\ C=\sum_{t,a,r}\frac{X_{tar}C_{tar}}{B_{tar}}\;E=\sum_{t,a,r}B_{tar}\\ F=\sum_{t,a,r}C_{tar} \end{array}$$ (29)

The estimates are then

$$\hat{\alpha }=\frac{CE-BF}{AE-B^{2}}$$ (30)

and

$$\hat{\mu }=\frac{CB-AE}{B^{2}-AE}$$ (31)

### Conditional expectations (step 4 in EM algorithm)

Now we will calculate the expected number phages conditional on the size of the burst $E\left(B_{tar}|C_{tar},\hat{\alpha },\hat{\mu },{\hat{\sigma }}^{2}\right)$ as well as the expected sum of lysis times given the burst size $E\left(X_{tar}|C_{tar},\hat{\alpha },\hat{\mu },{\hat{\sigma }}^{2}\right)$. Throughout this section, assay *a* and well *r* will be fixed; for notational convenience we will drop those subscripts and just denote $C_{tar},B_{tar},T_{iar},N_{ar},and\;X_{tar}$ by $C_{t},B_{t},T_{i},N,and\;X_{t},$ respectively. Because we will be simulating the joint distribution of $B_{t},X_{t}$ conditional on $C_{t}$ we will sometimes use a second subscript. In this case $B_{tj},X_{tj},T_{ij},N_{j}$ will represent the *j*th simulated value. To do this, we will first need the joint distribution of $X_{t}$ and $B_{t}$, which we obtain through the following simulation algorithm.

1. Simulate $N_{j}$ from a Poisson distribution with mean $\beta_{a}$. If $N_{j}=0$ repeat, otherwise proceed.
2. Simulate $T_{1j},T_{2j},\cdots ,T_{N_{j},j}$ from a shifted exponential with mean $\hat{\lambda }$, and shift ${\hat{t}}_{0}$ estimated using the likelihood equation (4).
3. Calculate $B_{tj}=\sum_{i=1}^{N_{j}}I\left\{T_{ij}\leq t\right\}$ and $X_{tj}=\sum_{i=1}^{T_{j}}T_{ij}I\left\{T_{ij}\leq t\right\}$ for each value of *t* in the assay. Round values of $X_{tj}$ to the nearest tenth of a minute (for values < 1 min), the nearest quarter minute for values between 1 min and 5 min, and the nearest minute for values between 5 min and 100 min. Store.
4. Repeat 1 through 3 for j=1,2,⋯,M where we set M=100,000.
5. For each value of *t*, convert the observed joint values of $\left(B_{tj},X_{tj}\right)$ to proportions.

The above algorithm generates the empirical distribution $f_{B_{t},X_{t}}\left(b_{t},x_{t}|\text{λ},t_{0}\right)$. We note that the distribution for $C_{t}$ conditional on $B_{t}$ and $X_{t}$ is normally distributed with mean $\text{α}X_{t}+\text{μ}B_{t}$ and variance $B_{t}{\text{σ}}^{2}$. Denote this distribution by

$$f_{C_{t}}\left(c_{t}|b_{t},x_{t},\alpha ,\mu ,\sigma^{2}\right)=\frac{1}{\sqrt{2\pi b_{t}}\sigma }\exp\left\{{\left(c_{t}-\alpha x_{t}-\mu b_{t}\right)}^{2}/\left(2b_{t}\sigma \right)\right\}$$ (32)

then

$$E\left(X_{t}|C_{t},\alpha ,\mu ,\sigma^{2}\right)=\sum_{x_{t}}\sum_{b_{t}}\frac{x_{t}f_{B_{t},X_{t}}\left(b_{t},x_{t}|\lambda ,t_{0}\right)f_{C_{t}}\left(c_{t}|b_{t},x_{t},\alpha ,\mu ,\sigma^{2}\right)}{f_{C_{t}}\left(c_{t}\right)}$$ (33)

and

$$E\left(B_{t}|C_{t},\alpha ,\mu ,\sigma^{2}\right)=\sum_{x_{t}}\sum_{b_{t}}\frac{b_{t}f_{B_{t},X_{t}}\left(b_{t},x_{t}|\lambda ,t_{0}\right)f_{C_{t}}\left(c_{t}|b_{t},x_{t},\alpha ,\mu ,\sigma^{2}\right)}{f_{C_{t}}\left(c_{t}\right)}$$ (34)

where

$$f_{C_{t}}\left(c_{t}\right)=\sum_{x_{t}}\sum_{b_{t}}f_{C_{t}}\left(c_{t}|b_{t},x_{t},\alpha ,\mu ,\sigma^{2}\right)f_{B_{t},X_{t}}\left(b_{t},x_{t}|\lambda ,t_{0}\right)$$ (35)

Using Equations 33 and 34 we can calculate the expected values of $X_{\text{tar}}$ and $B_{\text{tar}}$ for use in the EM algorithm.

## References

[bib1] Abedon S. T., 1989 Selection for bacteriophage latent period length by bacterial density: a theoretical examination. Microb. Ecol. 18: 79–88. 24196124 10.1007/BF02030117

[bib2] Abedon S. T., 1992 Lysis of lysis-inhibited bacteriophage T4-infected cells. J. Bacteriol. 174: 8073–8080. 1459956 10.1128/jb.174.24.8073-8080.1992 PMC207546

[bib3] Abedon S. T. Herschler T. D. Stopar D., 2001 Bacteriophage latent-period evolution as a response to resource availability. Appl. Environ. Microbiol. 67: 4233–4241. 11526028 10.1128/AEM.67.9.4233-4241.2001 PMC93152

[bib4] Afshinnekoo E. Meydan C. Chowdhury S. Jaroudi D. Boyer C., 2015 Geospatial resolution of human and bacterial diversity with city-scale metagenomics. Cell Syst. 1: 1–15. 10.1016/j.cels.2015.01.001 PMC4651444 26594662

[bib5] Barrell B. G. Air G. M. Hutchison C. A. III, 1976 Overlapping genes in bacteriophage φX174. Nature 264: 34–41. 1004533 10.1038/264034a0

[bib6] Bernal R. A. Hafenstein S. Esmeralda R. Fane B. A. Rossmann M. G., 2004 The φX174 protein J mediates DNA packaging and viral attachment to host cells. J. Mol. Biol. 337: 1109–1122. 15046981 10.1016/j.jmb.2004.02.033

[bib7] Bernhardt T. G. Roof W. D. Young R., 2002 The *Escherichia coli* FKBP-type PPIase SlyD is required for the stabilization of the E lysis protein of bacteriophage φX174. Mol. Microbiol. 45: 99–108. 12100551 10.1046/j.1365-2958.2002.02984.x

[bib8] Brown C. J. Zhao L. Evans K. J. Ally D. Stancik A. D., 2010 Positive selection at high temperature reduces gene transcription in the bacteriophage φX174. BMC Evol. Biol. 10: 378. 21129199 10.1186/1471-2148-10-378 PMC3003669

[bib9] Brown C. J. Stancik A. D. Roychoudhury P. Krone S. M., 2013 Adaptive regulatory substitutions affect multiple stages in the life cycle of the bacteriophage φX174. BMC Evol. Biol. 13: 1–12. 23506096 10.1186/1471-2148-13-66 PMC3608072

[bib10] Bull J. J., 2006 Optimality models of phage life history and parallels in disease evolution. J. Theor. Biol. 241: 928–938. 16616205 10.1016/j.jtbi.2006.01.027

[bib11] Bull J. J. Pfennig D. W. Wang I.-N., 2004 Genetic details, optimization and phage life histories. Trends Ecol. Evol. 19: 76–82. 16701232 10.1016/j.tree.2003.10.008

[bib12] Bull J. J. Millstein J. Orcutt J. Wichman H., 2006 Evolutionary feedback mediated through population density, illustrated with viruses in chemostats. Am. Nat. 167: E39–E51. 16670974 10.1086/499374

[bib13] Bull J. J. Heineman R. H. Wilke C. O., 2011 The phenotype-fitness map in experimental evolution of phage. PLoS One 6: e27796. 22132144 10.1371/journal.pone.0027796 PMC3222649

[bib14] Bull J. J. Vegge C. S. Schmerer M. Chaudhry W. N. Levin B. R., 2014 Phenotypic resistance and the dynamics of bacterial escape from phage control. PLoS One 9: e94690. 24743264 10.1371/journal.pone.0094690 PMC3990542

[bib15] Burnet F., 1929 A method for the study of bacteriophage multiplication in broth. Br. J. Exp. Pathol. 10: 109–115.

[bib16] Chao L. Rang C. U. Wong L. E., 2002 Distribution of spontaneous mutants and inferences about the replication mode of the RNA bacteriophage φ6. J. Virol. 76: 3276–3281. 11884552 10.1128/JVI.76.7.3276-3281.2002 PMC136006

[bib17] Cherwa J. E. Jr Sanchez-Soria P. Members of the University of Arizona Virology Laboratory Course 2007 Wichman H. A. Fane B. A., 2009 Viral adaptation to an antiviral protein enhances the fitness level to above that of the uninhibited wild type. J. Virol. 83: 11746–11750. 19726521 10.1128/JVI.01297-09 PMC2772694

[bib18] Dean A. M. Thornton J. W., 2007 Mechanistic approaches to the study of evolution: the functional synthesis. Nat. Rev. Genet. 8: 675–688. 17703238 10.1038/nrg2160 PMC2488205

[bib19] Delbrück M., 1945 The burst size distribution in the growth of bacterial viruses (bacteriophages). J. Bacteriol. 50: 131–135. 10.1128/JB.50.2.131-135.1945 20989330

[bib20] Denhardt D. T. Sinsheimer R. L., 1965 The process of infection with bacteriophage φX174. III. Phage maturation and lysis after synchronized infection. J. Mol. Biol. 12: 641–646. 5323480 10.1016/s0022-2836(65)80318-7

[bib21] Dennehy J. J. Wang I.-N., 2011 Factors influencing lysis time stochasticity in bacteriophage *λ*. BMC Microbiol. 11: 174. 21810267 10.1186/1471-2180-11-174 PMC3166277

[bib22] Fane B. A. Brentlinger K. L. Burch A. D. Chen M. Hafenstein S., 2006 X174 *et al.*, the *Microviridae*, pp. 129–145 in The Bacteriophages, Ed. 2, Chap. 11., edited by Calendar R. Abedon S. T. Oxford University Press, Oxford.

[bib23] Hadas H. Einav M. Fishov I. Zaritsky A., 1997 Bacteriophage T4 development depends on the physiology of its host *Escherichia coli*. Microbiology 143: 179–185. 9025292 10.1099/00221287-143-1-179

[bib24] Harms M. J. Thornton J. W., 2013 Evolutionary biochemistry: revealing the historical and physical causes of protein properties. Nat. Rev. Genet. 14: 559–571. 23864121 10.1038/nrg3540 PMC4418793

[bib25] Hayashi M. Aoyama A. Richardson D. L. Hayashi M. N., 1988 Biology of the bacteriophage φX174, pp. 1–71 in The Bacteriophages, Vol. 2, edited by Calendar R., Plenum Press, New York, NY.

[bib26] Hubbarde J. E. Wild G. Wahl L. M., 2007 Fixation probabilities when generation times are variable: the burst-death model. Genetics 176: 1703–1712. 17483420 10.1534/genetics.107.072009 PMC1931549

[bib27] Hutchison C. A. Sinsheimer R. L., 1963 Kinetics of bacteriophage release by single cells of φX174-infected *E. coli*. J. Mol. Biol. 7: 206–208. 14062652 10.1016/s0022-2836(63)80046-7

[bib28] Hutchison C. A. Sinsheimer R. L., 1966 The process of infection with bacteriophage φX174: X. Mutations in a φX lysis gene. J. Mol. Biol. 18: 429–447. 5968177 10.1016/s0022-2836(66)80035-9

[bib29] Hyman, P., and S. T. Abedon, 2009 Practical methods for determining phage growth parameters, pp. 175–202 in *Bacteriophages: Methods and Protocols*, *Vol. 1: Isolation*, *Characterization*, *and Interactions*, Vol. 501 of *Methods in Molecular Biology*, edited by M. R. J. Clokie, and A. M. Kropinski. Humana Press, Clifton, New Jersey. 10.1007/978-1-60327-164-6_18 19066822

[bib30] Labrie S. J. Dupuis M.-E. Tremblay D. M. Plante P.-L. Corbeil J., 2014 A new microviridae phage isolated from a failed biotechnological process driven by *Escherichia coli*. Appl. Environ. Microbiol. 80: 6992–7000. 25192988 10.1128/AEM.01365-14 PMC4249002

[bib31] McDonald J., 2014 Handbook of Biological Statistics, Ed. 3 Sparky House Publishing, Baltimore, MD.

[bib32] Mendel S. Holburn J. M. Schouten J. A. Bugg T. D. H., 2006 Interaction of the transmembrane domain of lysis protein E from bacteriophage φX174 with bacterial translocase MraY and peptidyl-prolyl isomerase SlyD. Microbiology 152: 2959–2967. 17005977 10.1099/mic.0.28776-0

[bib33] Meng X.-L. Rubin D. B., 1993 Maximum likelihood estimation via the ECM algorithm: a general framework. Biometrika 80: 267–268.

[bib34] Michel A. Clermont O. Denamur E. Tenaillon O., 2010 Bacteriophage φX174’s ecological niche and the flexibility of its *Escherichia coli* lipopolysaccharide receptor. Appl. Environ. Microbiol. 76: 7310–7313. 20833781 10.1128/AEM.02721-09 PMC2976268

[bib35] Newbold J. E. Sinsheimer R. L., 1970 Process of infection with bacteriophage φX174, XXXII. Early steps in the infection process: attachment, eclipse, and DNA penetration. J. Mol. Biol. 49: 49–66. 5450517 10.1016/0022-2836(70)90375-x

[bib36] Patwa Z. Wahl L. M., 2008 Fixation probability for lytic viruses: the attachment-lysis model. Genetics 180: 459–470. 18757918 10.1534/genetics.108.090555 PMC2535696

[bib37] Patwa Z. Wahl L. M., 2009 The impact of host-cell dynamics on the fixation probability for lytic viruses. J. Theor. Biol. 259: 799–810. 19464301 10.1016/j.jtbi.2009.05.008

[bib38] Pearson W. Wood T. Zhang Z. Miller W., 1997 Comparison of DNA sequences with protein sequences. Genomics 46: 24–36. 9403055 10.1006/geno.1997.4995

[bib39] Pepin K. M. Samuel M. A. Wichman H. A., 2006 Variable pleiotropic effects from mutations at the same locus hamper prediction of fitness from a fitness component. Genetics 172: 2047–2056. 16361237 10.1534/genetics.105.049817 PMC1456406

[bib40] Rabinovitch A. Hadas H. Einav M. Melamed Z. Zaritsky A., 1999 Model for bacteriophage T4 development in *Escherichia coli*. J. Bacteriol. 181: 1677–1683. 10049403 10.1128/jb.181.5.1677-1683.1999 PMC93561

[bib41] Rabinovitch A. Fishov I. Hadas H. Einav M. Zaritsky A., 2002 Bacteriophage T4 development in *Escherichia coli* is growth rate dependent. J. Theor. Biol. 216: 1–4. 12076123 10.1006/jtbi.2002.2543

[bib42] Roff D., 2002 Life History Evolution, Sinauer, Sunderland, MA.

[bib43] Roof W. Fang H. Young K. Sun J. Young R., 1997 Mutational analysis of *SlyD*, an *Escherichia coli* gene encoding a protein of the FKBP immunophilin family. Mol. Microbiol. 25: 1031–1046. 9350861 10.1046/j.1365-2958.1997.5201884.x

[bib44] Sanger F. Air G. M. Barrell B. G. Brown N. L. Coulson A. R., 1977 Nucleotide sequence of bacteriophage φX174 DNA. Nature 265: 687–695. 870828 10.1038/265687a0

[bib45] Shao Y. Wang I.-N., 2008 Bacteriophage adsorption rate and optimal lysis time. Genetics 180: 471–482. 18757924 10.1534/genetics.108.090100 PMC2535697

[bib46] Singh A. Dennehy J. J., 2014 Stochastic holin expression can account for lysis time variation in the bacteriophage *λ*. J. R. Soc. Interface 11: 20140140. 10.1098/rsif.2014.0140 PMC4006253 24718449

[bib47] Stearns S., 1992 The Evolution of Life Histories, Oxford University Press, Oxford.

[bib48] Stearns S. Ackermann M. Doebeli M. Kaiser M., 2000 Experimental evolution of aging, growth, and reproduction in fruitflies. Proc. Natl. Acad. Sci. USA 97: 3309–3313. 10716732 10.1073/pnas.060289597 PMC16235

[bib49] Storms Z. J. Sauvageau D., 2014 Evidence that the heterogeneity of a T4 population is the result of heritable traits. PLoS One 9: e116235. 25551763 10.1371/journal.pone.0116235 PMC4281060

[bib50] Storms Z. J. Brown T. Cooper D. G. Sauvageau D. Leask R. L., 2014 Impact of the cell life-cycle on bacteriophage T4 infection. FEMS Microbiol. Lett. 353: 63–68. 24822278 10.1111/1574-6968.12402

[bib51] Tanaka S. Clemons W. M. Jr, 2012 Minimal requirements for inhibition of MraY by lysis protein E from bacteriophage φX174. Mol. Microbiol. 85: 975–985. 22742425 10.1111/j.1365-2958.2012.08153.x PMC3429702

[bib52] van Elsas J. D. Semenov A. V. Costa R. Trevors J. T., 2011 Survival of *Escherichia coli* in the environment: fundamental and public health aspects. ISME J. 5: 173–183. 20574458 10.1038/ismej.2010.80 PMC3105702

[bib53] Wahl L. M. DeHaan C. S., 2004 Fixation probability favors increased fecundity over reduced generation time. Genetics 168: 1009–1018. 15514071 10.1534/genetics.104.029199 PMC1448855

[bib54] Wang I.-N., 2006 Lysis timing and bacteriophage fitness. Genetics 172: 17–26. 16219778 10.1534/genetics.105.045922 PMC1456144

[bib55] Wang I.-N. Dykhuizen D. E. Slobodkin L. B., 1996 The evolution of phage lysis timing. Evol. Ecol. 10: 545–558.

[bib56] Wichman H. A. Brown C. J., 2010 Experimental evolution of viruses: *Microviridae* as a model system. Phil. Trans. R. Soc. B 365: 2495–2501. 20643739 10.1098/rstb.2010.0053 PMC2935103

[bib57] Witte A. Brand E. Mayrhofer P. Narendja F. Werner L., 1998 Mutations in cell division proteins FtsZ and FtsA inhibit φX174 protein-E-mediated lysis of *Escherichia coli*. Arch. Microbiol. 170: 259–268. 9732440 10.1007/s002030050641

[bib58] Yin H. Marshall D., 2012 Microfluidics for single cell analysis. Curr. Opin. Biotechnol. 23: 110–119. 22133547 10.1016/j.copbio.2011.11.002

[bib59] Young R. Wang I.-N., 2006 Phage lysis, pp. 104–125 in The Bacteriophages, Ed. 2 chap 10., edited by Calendar R. Abedon S. T. Oxford University Press, Oxford.

[bib60] Young R. Wang I.-N. Roof W. D., 2000 Phages will out: strategies of host cell lysis. Trends Microbiol. 8: 120–128. 10707065 10.1016/s0966-842x(00)01705-4

[bib61] Zheng Y. Struck D. K. Dankenbring C. A. Young R., 2008 Evolutionary dominance of holin lysis systems derives from superior genetic malleability. Microbiology 154: 1710–1718. 18524925 10.1099/mic.0.2008/016956-0 PMC5995320

[bib62] Zheng Y. Struck D. K. Young R., 2009 Purification and functional characterization of φX174 lysis protein E. Biochemistry 48: 4999–5006. 19379010 10.1021/bi900469g PMC3100163

