# Supplementary material for: Genetically Determined Variation in Lysis Time Variance in the Bacteriophage φX174
Source: G3 (Bethesda). 2016 Feb 22;6(4):939–55. doi: 10.1534/g3.115.024075 (PMC4825663; doi:10.1534/g3.115.024075)
Supplement: Supporting Information [file supp_6_4_939__index.html]

Genetically Determined Variation in Lysis Time Variance in the Bacteriophage φX174 — Supporting Information 

# Genetically Determined Variation in Lysis Time Variance in the Bacteriophage φX174

## Supporting Materials for Baker *et al.*, 2016

**Files in this Data Supplement:**

- File S1 - Data and analysis code with commentary. (.zip, 3 MB)
