# Supplementary material for: Genetically Determined Variation in Lysis Time Variance in the Bacteriophage φX174
Source: G3 (Bethesda). 2016 Feb 22;6(4):939–55. doi: 10.1534/g3.115.024075 (PMC4825663; doi:10.1534/g3.115.024075)
Supplement: Supporting Information [file supp_g3.115.024075_G3_2015_024075-SI.zip › Lysis_Time_RMarkdown/Lysis_Time_RMarkdown_3.html]

RMarkdown File for Genetically determined variation in lysis time variance in the bacteriophage \phiX174 by Baker et al.


# RMarkdown File for *Genetically determined variation in lysis time variance in the bacteriophage \(\phi\)X174* by Baker et al.

#### *Craig Miller (crmiller@uidaho.edu)*

## Summary

This Markdown document and the accompaning source file (“Analysis\_Functions.R”) contains all the code to perform the analysis in Baker et al. *Genetically determined variation in lysis time variance in the bacteriophage \(\phi\)X174*. A lot of this analysis was done with brute force methods: maximum likelihoods were found by gridding parameter space, distributions for signficance tests were obtained by simulating data under the null and analyzing it under both the null and the alternative models. The result is that the analysis takes a long time to run (weeks on my desktop). There are booleans at the very beginning that allow control over what analysis is actually done. When the boolean for a section is set to FALSE, I instead read the results in from file and print summary tables and plots. Feel free to contact me if you have questions or run into problems.

### A note about file structure

I am assuming that the analysis is setup under “/Lysis\_Time\_RMarkdown”. Notice that the first lines of code below involve specifying pathways. The key file with all the functions drawn upon here is “Analysis\_Functions.R”. The raw data is found in “Raw Data (R-ready, txt)”. Note that within the data folder is a “READ ME.txt” file that provides a brief explanation of the data structure. The Weibull tests for a hazard with rate 1 (exponential) is found in the “Weibull LRT” folder; the analysis of \(\lambda\) and \(t\_0\) occurs within the “Lambda\_t0” folder; the regression analysis to determine how burst size relates to time is found in “Burstsize\_regression”. The appropriate folder and file structure already exist in the zip file available for download. This file also contains all the distributions and parameter estimate files which amount to the analysis output.

### User Defined Settings & Load Packages

```
  base.path <- "/Users/craigmiller/Work/R_Programming/Weinreich/New_2015/Lysis_time_RMarkdown/"
  data.path <- paste(base.path, "Raw Data (R-ready, txt)/", sep="")  # Specify data directly
  lambdat0.path <- paste(base.path, "Lambda_t0/", sep="")  # Specify folder where lambda and t0 analysis occurs
  bustsize.path <- paste(base.path, "Burstsize_regression/", sep="")  # Specify folder where burst size by time regression occurs
  source.path <- paste(base.path, "Analysis_Functions.R", sep="")  # Specify where all the analysis functions reside
  source(file=source.path)
  weibull.path <- paste(base.path, "Weibull LRT/", sep="")
  burst.cutoff <- 7          #counts > burst.cutoff are treated as burst events, <= burst.cutoff are considered unburst wells
  n.weibull.boots <- 100       # number of bootstraps to use in Weibull LRT for null model of alpha=1 (vs alpha != 1).
  run.weibull.LRT.analysis <- FALSE    # Takes many hours. If FALSE, we just read in the distributions (from simulations) to illustrate results
  run.lambdat0.estimation <- FALSE      # Time consuming. If FALSE, results are read in. If TRUE, conducts analysis.
  run.lambdat0.CIestimation <- FALSE 
  run.pairwise.lambdat0.tests <- FALSE   # pairwise test for any diffrence b/t clones (either in lambda, t.0 or both)
  run.pairwise.lambda.tests <- FALSE    # pairwise test for difference in lambda only (t.0 free to vary)
  run.pairwise.t0.tests <- FALSE        # pairwise test for difference in t.0 (lambda free to vary)
  n.lambdat0.boots <- 100               # Number of bootstrap simulations to use for calculating confidence intervals. 
  n.pairwist.lambdat0.boots <- 100      # Number of bootstraps to use in pairwise tests for differences in lambda and t0 b/t clones
  run.burstsize.regression <- FALSE     # boolean controls whether to run the burstsize regression or not.
  n.em.iterations <- 500                # number of em iterations in the EM algorithm.          
  
  lambda.v <- seq(4,18, by=0.1)   # was seq(1, 25)
  lambda.v.boot <- seq(4, 18, by=0.1)
    t.0.v <- seq(6,15, by=0.1)
    t.0.v.boot <- seq(8, 16, by=0.2)
  
  library(xtable)
```

### Read in Data

```
  #=== Read in Data and define some basic parameters like mutant names and the number of them ===
  
  setwd(data.path)
  data.WT <- read.table(file="WT_data.txt", header=TRUE)
    data.319 <- read.table(file="319_data.txt", header=TRUE)
    data.321 <- read.table(file="321_data.txt", header=TRUE)
    data.323 <- read.table(file="323_data.txt", header=TRUE)
    data.324 <- read.table(file="324_data.txt", header=TRUE)
    data.5 <- read.table(file="5_data.txt", header=TRUE)
    data.6 <- read.table(file="6_data.txt", header=TRUE)
    data.4B <- read.table(file="4B_Data rounded.1.txt", header=TRUE)
    data <- list(WT=data.WT, m.319=data.319, m.321=data.321, m.323=data.323, m.324=data.324, m.5=data.5, m.6=data.6, m.4B=data.4B)
    mutant.v <- c("WT", "m.319", "m.321", "m.323","m.324", "m.5", "m.6", "m.4B")
    n.muts <- length(mutant.v)
    beta.by.day.list <- vector(mode="list", length=length(mutant.v))
```

### Estimate \(\beta\) parameters

The number of phage in each well is assumed to be Poisson distributed with mean \(\beta\). This \(\beta\) may differ for each assay and each mutant. Here we use the data to estimate \(\beta\) (using equation 2 from the paper) and then append this on to the data matrix (data). The data is packaged up in a list (data.by.mut) that we access in subsequent anlayses.

```
  beta.by.day.list <- vector(mode="list", length=n.muts)
    names(beta.by.day.list) <- mutant.v
    data.by.mut <- vector("list", n.muts)
    
    for (mut.i in 1:length(mutant.v)){
      mutant <- mutant.v[mut.i]
      new.tiger.data <- data[[mut.i]]
      
      # === Create matricies to hold info (filled below) ===
        all.data <- matrix(nrow=1, ncol=6)              # new data is matrix where all observations are concatenated--1 row per well/plate
        colnames(all.data) <- c("burst", "time", "beta", "date", "batch", "dataset")   # burst=burst size...batch is identifier in case > 1 datasets are done on same day using different stock
        all.data <- as.data.frame(all.data)
        dates <- colnames(new.tiger.data)               # get assay dates from column names
        dates <- dates[-1]                             # remove 1st column header (time)
        n.days <- length(dates)                        # number of days
        time.v <- new.tiger.data[,1]
        beta.by.day <- matrix(nrow=1, ncol=n.days)
        colnames(beta.by.day) <- dates
      
     # === Now the actual estimation loop ===
        for (day.i in 1:n.days){
            day.v.raw <- new.tiger.data[,day.i+1]   # b/c 1st column is time, data is right-shifted 1 column
            not.NA <- which(is.na(day.v.raw)==FALSE)
            day.v <- day.v.raw[not.NA]
                beta.by.day[day.i] <- round(est.beta.day.NEW(day.v)$beta.hat,4)
        }  # next day.i

       beta.by.day.list[[mut.i]] <- beta.by.day
       
       
     #   === Put data with beta estimtes into all.data
    for (day.i in 1:n.days){
        for (time.i in 1:length(time.v)){
            if (is.na(new.tiger.data[time.i, day.i+1])==FALSE){   # want to skip NA cells (no data)
                data.row <- rep(NA, length(all.data[1,]))
                data.row[1] <- new.tiger.data[time.i, day.i + 1]
                data.row[2] <- new.tiger.data[time.i, 1]            
                data.row[3]<- beta.by.day[day.i]
                data.row[4] <- dates[day.i]
                all.data <- rbind(all.data, data.row)
                }
            }
        }
    all.data <- all.data[-1,]
    
    data.by.mut[[mut.i]] <- all.data
       
    }   # next mut.i
    
    # The structure of the data 
  head(data.by.mut[[mut.i]],10)
```

```
##    burst time  beta        date batch dataset
## 2      0    5 0.468 March_18_12  <NA>    <NA>
## 3      0 5.25 0.468 March_18_12  <NA>    <NA>
## 4      0  5.5 0.468 March_18_12  <NA>    <NA>
## 5      2 5.75 0.468 March_18_12  <NA>    <NA>
## 6      0    6 0.468 March_18_12  <NA>    <NA>
## 7      0 6.25 0.468 March_18_12  <NA>    <NA>
## 8      0  6.5 0.468 March_18_12  <NA>    <NA>
## 9      0 6.75 0.468 March_18_12  <NA>    <NA>
## 10     2    7 0.468 March_18_12  <NA>    <NA>
## 11     0 7.25 0.468 March_18_12  <NA>    <NA>
```

### Conduct Likelihood Ratio Test for Weibull Parameter \(\alpha=1\)

Under the Weibull \(\alpha=1\) means that the probability of an event (lysis in our case) is constant per unit time. We first wish to know whether the data is consistent with this simple model. We do this by defining the null as \(\alpha=1\), calculating the likelihood of the data under this null and under an alternative where \(\alpha\) is esitimated, taking the ratio, and then asking is this observed ratio unusual when the null is true. We get the distribution of the ratio under the null by simulation. Note that this code is slow as the likelihood search is by brute force. Setting run.weibull.LRT.analysis <- TRUE means full analysis will be done; setting it to FALSE reads in the results and plots.

```
    LR.null.dist.list <- vector(mode="list", length=n.muts)
    LR.obs.list <- vector(mode="list", length=n.muts)
    r.list <- vector(mode="list", length=n.muts)
    boot.parms.by.mut <- vector("list", n.muts)
    setwd(weibull.path)
    
    if (run.weibull.LRT.analysis == TRUE){    # Calculate LR and distributions
      for (mut.i in 1:n.muts){
       all.data <- data.by.mut[[mut.i]]
       r <- est.burst.prob.parms.for.weibullLRT(all.data)
       LR.obs <- r$LR
       t.0.null <- r$t.0.null
       lam.null <- r$lam.null
       alph.null <- 1   
      
       LR.obs.list[[mut.i]] <- LR.obs
       r.list[[mut.i]] <- unlist(r)
    
       LR.null.dist <- rep(NA, n.weibull.boots)
       parms.by.boot <- matrix(nrow=n.weibull.boots, ncol=length(unlist(r)))
       colnames(parms.by.boot) <- names(unlist(r))
     
       for (boot.i in 1:n.weibull.boots){
          print(paste("bootstrap rep.i=", boot.i))
          sim.data.null <- sim.dataset(lam.null, alph.null, t.0.null, all.data)
          r.boot <- est.burst.prob.parms.for.weibullLRT(sim.data.null)
          LR.null.dist[boot.i] <- r.boot$LR
          parms.by.boot[boot.i,] <- unlist(r.boot)
      }
    
      LR.null.dist.list[[mut.i]] <- LR.null.dist
      boot.parms.by.mut[[mut.i]] <- parms.by.boot
      
      write.table(LR.null.dist, file=paste(mutant, ".Weibull.LR.null.dist(new).txt", sep=""), row.names=F, col.names=F, sep="\t")
        write.table(t(unlist(r)), file=paste(mutant, ".Weibull.LR.results(new).txt", sep=""), row.names=F, col.names=T, sep="\t")   
    }  #next mut.i
  } else{
    weibull.results <- vector("list", n.muts)
    for (mut.i in 1:n.muts){
      LR.null.dist.list[[mut.i]] <- read.table(file=paste(mutant.v[mut.i], ".Weibull.LR.null.dist.txt", sep=""), header=FALSE, sep="")
      weibull.results[[mut.i]] <- read.table(file=paste(mutant.v[mut.i], ".Weibull.LR.results.txt", sep=""), header=TRUE, sep="")
    }
  }  
  
  for (mut.i in 1:n.muts){
    LR.obs <- weibull.results[[mut.i]]$LR
    p.value <- length(which(t(LR.null.dist.list[[mut.i]])>LR.obs))/length(t(LR.null.dist.list[[mut.i]]))
    LR.under.null <- t(LR.null.dist.list[[mut.i]])
    LR.under.null[which(LR.under.null <0)] <- 0     # few just under zero b/c of granular resolution of likelihood calculation.
    breaks <- seq(0,8, by=0.25)
    hist(LR.under.null, xlab="Likelihood Ratio Score", main=paste(mutant.v[mut.i], ": Distribution of Likelihood Ratio Under Null"), breaks=breaks)
    points(weibull.results[[mut.i]]$LR, 0, pch=23, bg="red", cex=2)
      legend("topright", bty="n", pch=c(23,NA), legend=c("Observed LR", paste("p=", p.value)), pt.bg="red", cex=1.5)
  }
```

The red diamonds in the histograms show you that in no case is the observed likehood ratio in the tail of the null distribution. Therefore, we adopt the null model of \(\alpha=1\) for the rest of the work.

### Estimation of \(t\_0\) and \(\lambda\)

Equation 4 in the paper gives the log-likelihood of the data as a function of \(t\_0\) and \(\lambda\) jointly. This code conducts a brute force search across a grid of joint values. The range of values on that grid is specified by t.0.v and lambda.v. This is time consuming.

```
    # === Search ranges are input at top. But that input can be overwritten here if it's more convenient.
  #lambda.v <- seq(4,18, by=0.1)   # was seq(1, 25)
    #t.0.v <- seq(6,15, by=0.1)
    
  if (run.lambdat0.estimation == TRUE){
    for (mut.i in 1:n.muts){
      all.data <- data.by.mut[[mut.i]]
        t.0.by.lam <- matrix(nrow=length(t.0.v), ncol=length(lambda.v))
        burst.wells <- which(abs(as.numeric(all.data$burst))>burst.cutoff)   # note use of absolute value so overgrown plates are included as burst wells
      burst.times <- as.numeric(all.data$time[burst.wells])
      max.burst <- max(burst.times)
      min.burst <- min(burst.times)
      alpha <- 1
  
            for (t.0.i in 1:length(t.0.v)){
                print(paste("t.0=", t.0.v[t.0.i]))
                for (lam.i in 1:length(lambda.v)){
                        lambda <- lambda.v[lam.i]
                        t.0.by.lam[t.0.i, lam.i] <- calc.like.given.alph.lam.combo.data(alpha, lambda, t.0.v[t.0.i], all.data)$ln.Like
                    } #next lam.i
                }  #next t.0.i
  
      max.parms <- which(t.0.by.lam == max(t.0.by.lam, na.rm=TRUE), arr.ind = TRUE)
      max.log.likelihood <- t.0.by.lam[max.parms]
      alpha.hat <- 1
      lambda.hat <- lambda.v[max.parms[2]]
        t.0.hat <- t.0.v[max.parms[1]]
        burst.parm.estimates <- cbind(lambda.hat=lambda.hat, t.0.hat=t.0.hat)
        if (lambda.hat==lambda.v[1]){print("warning: lambda.hat at boundary of values searched")}
        if (lambda.hat==lambda.v[length(lambda.v)]){print("warning: lambda.hat at boundary of values searched")}
        if (t.0.hat==t.0.v[1]){print("warning: t.0.hat at boundary of values searched")}
        if (t.0.hat==t.0.v[length(t.0.v)]){print("warning: t.0.hat at boundary of values searched")}
        plot.w.t.and.f.burst(t.0.hat, lambda.hat, alpha.hat, all.data, mutant.v[mut.i])
        burst.parm.estimates.list[[mut.i]] <- burst.parm.estimates
    
        setwd(lambdat0.path)
        write.table(beta.by.day, file=paste(mutant.v[mut.i], ".beta.by.day.txt", sep=""), append=FALSE, sep="\t", col.names=TRUE, row.names=FALSE)
        write.table(burst.parm.estimates, file=paste(mutant.v[mut.i], ".lambda.t0.est.txt", sep=""), append=FALSE, sep="\t", col.names=TRUE, row.names=FALSE)
        write.table(max.log.likelihood, file=paste(mutant.v[mut.i], ".log.like.lambda.t0.txt", sep=""), append=FALSE, sep="\t", col.names=FALSE, row.names=FALSE)
        write.table(t.0.by.lam, file=paste(mutant, ".t.0.by.lam.txt", sep=""), append=FALSE, sep="\t", col.names=FALSE, row.names=FALSE)
        write.table(t.0.v, file=paste(mutant.v[mut.i], ".t.0.v.txt", sep=""), append=FALSE, sep="\t", col.names=FALSE, row.names=FALSE)
        write.table(lambda.v, file=paste(mutant.v[mut.i], ".lambda.v.txt", sep=""), append=FALSE, sep="\t", col.names=FALSE, row.names=FALSE)
  
    }  #next mut.i   # unpound to just get the point estimates
  } else{
    print("Note, lambda and t0 not estimated at runtime. Instead, values have been read-in from file.")
    lam.t0.table <- as.data.frame(matrix(nrow=n.muts, ncol=3))
    colnames(lam.t0.table) <- c("mutant", "lambda.hat", "t.0.hat")
    setwd(lambdat0.path)
    for (mut.i in 1:n.muts){
      est <- read.table(paste(mutant.v[mut.i], ".lambda.t0.est.txt", sep=""), header=TRUE)
      lam.t0.table[mut.i,] <- c(mutant.v[mut.i], est)
    }
    print(lam.t0.table)
  }
```

```
## [1] "Note, lambda and t0 not estimated at runtime. Instead, values have been read-in from file."
##   mutant lambda.hat t.0.hat
## 1     WT       11.2    12.2
## 2  m.319        5.8    12.7
## 3  m.321        4.8    14.5
## 4  m.323        6.2    13.9
## 5  m.324        6.0    13.4
## 6    m.5        5.7    13.2
## 7    m.6        8.9    10.0
## 8   m.4B        9.5    12.7
```

### Confidence bounds on \(t\_0\) and \(\lambda\) using parametric bootstrap

The idea here is to simulate datasets under the model parameter estimates and then analyze each dataset. From this we can get confidence intervals on \(t\_0\) and \(\lambda\) both indivdually and jointly. Be warned this is slow. If it takes 30 minutes to get point estimates and you do 100 bootstraps, it will take 100\*30 minutes=50 hours to do this analysis. The boolean run.lambdat0.CIestimation set at the top of this file let’s you opt in or out of this. If set to FALSE, then we just read the results in from file and print the table summary results.

```
  if (run.lambdat0.CIestimation == TRUE){
    for (mut.i in 1:n.muts){
      # Read in parameter estimates generated in last code block.   
      beta.by.day <- beta.by.day.list[[mut.i]] 
      burst.parm.estimates <- read.table(file=paste(mutant.v[mut.i], ".lambda.t0.est.txt", sep=""), header=TRUE)
      lambda.hat <- burst.parm.estimates$lambda.hat
      t.0.hat <- burst.parm.estimates$t.0.hat
      
      n.boots <-n.lambdat0.boots
          bootstrap.lambdas <- matrix(nrow=n.boots, ncol=2)
          colnames(bootstrap.lambdas) <- c("t.0.hat", "lambda.hat")
          bootstrap.maxloglikes <- rep(NA, n.boots)
          lambda.v <- lambda.v.boot
          t.0.v <- t.0.v.boot
          
          all.data <- data.by.mut[[mut.i]]
          burst.times <- as.numeric(all.data$time[burst.wells])
      max.burst <- max(burst.times)
      min.burst <- min(burst.times)
        
          for (rep.i in 1:n.boots){
            #print(paste("bootstrap rep.i=", rep.i))
            sim.data <- sim.dataset(lambda.hat, alpha.hat, t.0.hat, all.data)
            boot.parms <-  estimate.lambda.hat.t.0(sim.data, min.burst)
            bootstrap.lambdas[rep.i, 1] <- boot.parms[1]
            bootstrap.lambdas[rep.i, 2] <- boot.parms[2] 
            bootstrap.maxloglikes[rep.i] <- boot.parms[3]
          }
            
            bootstrap.lambdas <- as.data.frame(cbind(bootstrap.lambdas, lnLike=bootstrap.maxloglikes))
            sorted.bootstraps <- sort(bootstrap.lambdas[,2])
            n <- length(sorted.bootstraps)
            lambda.low <- sorted.bootstraps[round(n*0.025)]
            lambda.high <- sorted.bootstraps[round(n*0.975)]
            
            sorted.t.0.hats <- sort(bootstrap.lambdas[,1])
            t.0.low <- sorted.t.0.hats[round(n*0.025)]
            t.0.up <- sorted.t.0.hats[round(n*0.975)]
            
            boot.CIs <- rbind(cbind(point=lambda.hat, low=lambda.low, high=lambda.high), cbind(point=t.0.hat, low=t.0.low, high=t.0.up))
            rownames(boot.CIs) <- c("lambda", "t.0")
            boot.CIs <- as.data.frame(boot.CIs)
            
            mean.boot.lambda <- mean(bootstrap.lambdas[,2])
            mean.boot.t.0 <- mean(bootstrap.lambdas[,1])
            
            setwd(paste(lambdat0.path, "Bootstraps", sep="/"))
            write.table(boot.CIs, file=paste(mutant.v[mut.i], ".lambda.t0.CIs.txt", sep=""), append=FALSE, sep="\t", col.names=TRUE, row.names=TRUE)
        write.table(bootstrap.lambdas, file=paste(mutant.v[mut.i], ".boot.lambda.t0.txt", sep=""), append=FALSE, sep="\t", col.names=TRUE, row.names=FALSE)
    }
  } else{
    print("Note: not running code to estimate confidence intervals. Instead reading in result files.")
    setwd(paste(lambdat0.path, "Bootstraps", sep="/"))
    for (mut.i in 1:n.muts){
      est <-  read.table(file=paste(mutant.v[mut.i], ".lambda.t0.CIs.txt", sep=""))
      est <- cbind(mutant=rep(mutant.v[mut.i],2), est)
      rownames(est) <- paste(rownames(est), "_", mutant.v[mut.i], sep="")
      if (mut.i == 1){
        est.CI.table <- est
      } else{
        est.CI.table <- rbind(est.CI.table, est)
      }
    }
    print(est.CI.table)
  }
```

```
## [1] "Note: not running code to estimate confidence intervals. Instead reading in result files."
##              mutant point   low  high
## lambda_WT        WT  11.2  7.50 15.25
## t.0_WT           WT  12.2 11.00 13.25
## lambda_m.319  m.319   5.8  4.00  7.75
## t.0_m.319     m.319  12.7 12.00 13.00
## lambda_m.321  m.321   4.8  3.50  6.25
## t.0_m.321     m.321  14.5 14.00 14.75
## lambda_m.323  m.323   6.2  4.50  8.50
## t.0_m.323     m.323  13.9 13.25 14.25
## lambda_m.324  m.324   6.0  4.25  8.25
## t.0_m.324     m.324  13.4 12.50 13.50
## lambda_m.5      m.5   5.7  4.00  7.50
## t.0_m.5         m.5  13.2 12.50 13.50
## lambda_m.6      m.6   8.9  6.25 12.25
## t.0_m.6         m.6  10.0  9.00 10.75
## lambda_m.4B    m.4B   9.5  7.00 12.50
## t.0_m.4B       m.4B  12.7 11.75 13.00
```

### Test for differences in either \(t\_0\) and \(\lambda\) (or both) b/t pairs of clones

As the paper lays out, there are a few nulls to consider: both \(t\_0\) and \(\lambda\) the same between the pair of clones, \(t\_0\) the same but \(\lambda\) free to differ, and \(\lambda\) the same but \(t\_0\) free to differ. We begin with the null that both \(\lambda\) and \(t\_0\) are the same in the pairwise comparison.

```
    if (run.pairwise.lambdat0.tests == TRUE){
      n.boot.reps  <- n.pairwist.lambdat0.boots     
      
      for (mut.i in 1:(n.muts-1)){
          for (mut.j in (mut.i+1):n.muts){
            print(paste("===== mut.i=", mut.i, "   mut.j=", mut.j, " ====="))

            data.set.1 <- data[[mut.i]]
            data.set.2 <- data[[mut.j]]
    
            lambda.v <- seq(4, 20, by=0.5)   # bit wider and coarser than the point estimation procedure
            t.0.v <- seq(6, 16, by=0.5)

            preped.data.1 <- prep.data(data.set.1)
            all.data.1 <- preped.data.1$all.data
            dates.1 <- preped.data.1$dates
            beta.by.day.1 <- preped.data.1$beta.by.day
            burst.wells.1 <- preped.data.1$burst.wells
            burst.times.1 <- preped.data.1$burst.times
            max.burst.1 <- preped.data.1$max.burst 
        
            preped.data.2 <- prep.data(data.set.2)
            all.data.2 <- preped.data.2$all.data
            dates.2 <- preped.data.2$dates
            beta.by.day.2 <- preped.data.2$beta.by.day
            burst.wells.2 <- preped.data.2$burst.wells
            burst.times.2 <- preped.data.2$burst.times
            max.burst.2 <- preped.data.2$max.burst 
        
            print("Analyzing mut.i")
        
            lambda.results.1 <- find.lambda.hat(all.data.1, t.0.v, lambda.v)
            lambda.hat.1 <- lambda.results.1$lambda.hat
            t.0.hat.1 <- lambda.results.1$t.0.hat
            m.ln.like.1 <- lambda.results.1$max.log.likelihood
            lambda.1.CI <- lambda.results.1$lambda.CI
            t.0.1.CI <- lambda.results.1$t.0.CI
        
            print("Analyzing mut.j")
    
            lambda.results.2 <- find.lambda.hat(all.data.2, t.0.v, lambda.v)
            lambda.hat.2 <- lambda.results.2$lambda.hat
            t.0.hat.2 <- lambda.results.2$t.0.hat
            m.ln.like.2 <- lambda.results.2$max.log.likelihood
            lambda.2.CI <- lambda.results.2$lambda.CI
            t.0.2.CI <- lambda.results.2$t.0.CI
        
            print("Analyzing pooled mut.i + mut.j")
    
            all.data.12.comb <- rbind(all.data.1, all.data.2)
            lambda.results.12c <- find.lambda.hat(all.data.12.comb, t.0.v, lambda.v, min(c(burst.times.1, burst.times.2)))
            lambda.hat.12c <- lambda.results.12c$lambda.hat
            t.0.hat.12c <- lambda.results.12c$t.0.hat
            m.ln.like.12c <- lambda.results.12c$max.log.likelihood
            lambda.12.CI <- lambda.results.12c$lambda.CI
            t.0.12.CI <- lambda.results.12c$t.0.CI
    
            # Summarize our observed likelihoods and the ratio of alt to null (LR.obs)
            ln.L.alt <- m.ln.like.1 + m.ln.like.2
            ln.L.null <- m.ln.like.12c
            LR.obs <- ln.L.alt - ln.L.null   # This is your likelihood ratio. You should report this statistic when summarizing the analysis.
    
            setwd(paste(lambdat0.path, "Pairwise_tests", sep="/"))
            mut.1 <- mutant.v[mut.i]
            mut.2 <- mutant.v[mut.j]
            summary <- c(mut.1=mut.1, mut.2=mut.2, lambda.hat.1=lambda.hat.1, t.0.hat.1=t.0.hat.1, lambda.hat.2=lambda.hat.2, t.0.hat.2=t.0.hat.2, ln.L.1 = m.ln.like.1, ln.L.2=m.ln.like.2, ln.L.alt=ln.L.alt, ln.L.null=ln.L.null, LR.obs=LR.obs)
            summary <- as.data.frame(t(summary))
            write.table(summary, file=paste("muts.", mut.1, ".", mut.2, ".summary.txt", sep=""), col.names=TRUE, row.names=FALSE, sep="\t")
        
            # ==== Now we want the distribution of LR.obs when the null is true. ====
     
            log.results <- as.data.frame(matrix(nrow=n.boot.reps, ncol=6))
            colnames(log.results) <- c("ln.L.1.null", "ln.L.2.null", "ln.L.12.null", "ln.L.alt.boot", "ln.L.null.boot", "LR.null.boot")

            LR.boots <- rep(NA, n.boot.reps)
        
            for (boot.i in 1:n.boot.reps){
                print(paste("bootstrap rep.i=", boot.i))
                sim.data.1.null <- sim.dataset(lambda.hat.12c, alpha, t.0.hat.12c, all.data.1)
                sim.data.2.null <- sim.dataset(lambda.hat.12c, alpha, t.0.hat.12c, all.data.2)
                sim.data.12c.null <- rbind(sim.data.1.null, sim.data.2.null)
    
                lambda.results.1.null <- find.lambda.hat(sim.data.1.null, t.0.v, lambda.v)
                lambda.results.2.null <- find.lambda.hat(sim.data.2.null, t.0.v, lambda.v)
                lambda.results.12c.null <- find.lambda.hat(sim.data.12c.null, t.0.v, lambda.v)
                ln.L.1.null <- lambda.results.1.null$max.log.likelihood
                ln.L.2.null <- lambda.results.2.null$max.log.likelihood
                ln.L.12c.null <- lambda.results.12c.null$max.log.likelihood

                ln.L.alt.boot <- ln.L.1.null + ln.L.2.null
                ln.L.null.boot <- ln.L.12c.null
                LR.null.boot <- ln.L.alt.boot - ln.L.null.boot
    
                LR.boots[boot.i] <- LR.null.boot
            
                log.results$ln.L.1.null[boot.i] <- ln.L.1.null
                log.results$ln.L.2.null[boot.i] <- ln.L.2.null
                log.results$ln.L.12.null[boot.i] <- ln.L.12c.null
                log.results$ln.L.alt.boot[boot.i] <- ln.L.alt.boot
                log.results$ln.L.null.boot[boot.i] <- ln.L.null.boot
                log.results$LR.null.boot[boot.i] <- LR.null.boot
            }   # NEXT boot.i
        
            write.table(log.results, file=paste("muts.", mutant.v[mut.i], ".", mutant.v[mut.j], ".log.results.txt", sep=""), col.names=TRUE, row.names=FALSE, sep="\t")
        } # next mut.j
      } # next mut.i
    } else{
      print("Note: not running test for differences in lambda or t0. Instead reading results from file.")
    }
```

```
## [1] "Note: not running test for differences in lambda or t0. Instead reading results from file."
```

```
      p.val.m <- as.data.frame(matrix(nrow=n.muts, ncol=n.muts))
      colnames(p.val.m) <- mutant.v
      rownames(p.val.m) <- mutant.v
      setwd(paste(lambdat0.path, "Pairwise_tests", sep="/"))
            
      for (mut.i in 1:(n.muts-1)){
        mut.1 <- mutant.v[mut.i]
        for (mut.j in (mut.i+1):n.muts){
              mut.2 <- mutant.v[mut.j]
            smry <- read.table(file=paste("muts.", mut.1, ".", mut.2, ".summary.txt", sep=""), header=TRUE)
            log.res <- read.table(file=paste("muts.", mut.1, ".", mut.2, ".log.results.txt", sep=""), header=TRUE)
              n.reps <- length(log.res$LR.null.boot)
              lam.obs <- smry$LR.obs
              p.val.m[mut.j, mut.i] <- length(which(log.res$LR.null.boot > lam.obs))/n.reps
              p.val.m[mut.i, mut.j] <- ""
              p.val.m[mut.i, mut.i] <- ""
              p.val.m[mut.j, mut.j] <- ""
          }
      }
    write.table(p.val.m, file="mut_by_mut_lam_t0_p_values.txt", sep="\t", row.names=TRUE, col.names=TRUE)
    print(p.val.m)
```

```
##          WT m.319 m.321 m.323 m.324   m.5 m.6 m.4B
## WT                                                
## m.319 0.028                                       
## m.321 0.002 0.006                                 
## m.323 0.062 0.132 0.448                           
## m.324 0.028 0.548 0.134 0.698                     
## m.5    0.01 0.768 0.082 0.438 0.778               
## m.6   0.012 0.008     0     0     0 0.008         
## m.4B    0.8 0.058  0.01 0.192  0.14 0.076   0
```

Table shows pairwise p-values of null that both \(\lambda\) and \(t\_0\) are the same b/t the pair.

### Test for differences in \(\lambda\) b/t pairs of clones

```
    # =============== Now, a similar LRT where we ask if lambda is signficiantly different (regardless of t.0) =================
#   We'll do this by comapring two hypotheses: (1) lambda same for both mutants (t.0 free to differ)
#   (2) Lambda free to differ b/t mutants (and t.0. can as well)
#   How do we do this?  Iterate through lambda values (in lambda.v)
#   For each, iterate over t.0.v, calculating lnL of each dataset lnL.1|t.0 and lnL.2|t.0
#   Once all t.0 values are calculated, find best for each mutant: lnL.1 and lnL.2
#   Sum lnL.1 + lnL.2 = lnL.12|lam. For a fixed lambda, this is the best fit.
#   Also find global best for each mutant. Sum to get lnL under alt model.
#   Take differences: LRobs.
#   Simulate under null model and repeat calclations. Determine where LRobs falls in distribution.
    
    if (run.pairwise.lambda.tests == TRUE){
      n.boot.reps  <- n.pairwist.lambdat0.boots  
      p.vals.lam <- as.data.frame(matrix(nrow=n.muts, ncol=n.muts))
      colnames(p.vals.lam) <- mutant.v
      rownames(p.vals.lam) <- mutant.v
      
      for (mut.i in 1:(n.muts-1)){
          for (mut.j in (mut.i+1):n.muts){
            print(paste("===== mut.i=", mut.i, "   mut.j=", mut.j, " ====="))

            data.set.1 <- data[[mut.i]]
            data.set.2 <- data[[mut.j]]
            mutant.1 <- mutant.v[mut.i]
            mutant.2 <- mutant.v[mut.j]
    
    
            lambda.v <- seq(4, 20, by=0.5)   # bit wider and coarser than the point estimation procedure
            t.0.v <- seq(6, 16, by=0.5)

            preped.data.1 <- prep.data(data.set.1)
            all.data.1 <- preped.data.1$all.data
            dates.1 <- preped.data.1$dates
            beta.by.day.1 <- preped.data.1$beta.by.day
            burst.wells.1 <- preped.data.1$burst.wells
            burst.times.1 <- preped.data.1$burst.times
            max.burst.1 <- preped.data.1$max.burst 
        
            preped.data.2 <- prep.data(data.set.2)
            all.data.2 <- preped.data.2$all.data
            dates.2 <- preped.data.2$dates
            beta.by.day.2 <- preped.data.2$beta.by.day
            burst.wells.2 <- preped.data.2$burst.wells
            burst.times.2 <- preped.data.2$burst.times
            max.burst.2 <- preped.data.2$max.burst 
          
            print("Analyzing mut.i")
        
            lambda.results.1 <- find.lambda.hat(all.data.1, t.0.v, lambda.v)
            lambda.hat.1 <- lambda.results.1$lambda.hat
            t.0.hat.1 <- lambda.results.1$t.0.hat
            m.ln.like.1 <- lambda.results.1$max.log.likelihood
            lambda.1.CI <- lambda.results.1$lambda.CI
            t.0.1.CI <- lambda.results.1$t.0.CI
            t.0.by.lam.1 <- lambda.results.1$t.0.by.lam
        
            print("Analyzing mut.j")
    
            lambda.results.2 <- find.lambda.hat(all.data.2, t.0.v, lambda.v)
            lambda.hat.2 <- lambda.results.2$lambda.hat
            t.0.hat.2 <- lambda.results.2$t.0.hat
            m.ln.like.2 <- lambda.results.2$max.log.likelihood
            lambda.2.CI <- lambda.results.2$lambda.CI
            t.0.2.CI <- lambda.results.2$t.0.CI
            t.0.by.lam.2 <- lambda.results.2$t.0.by.lam
        
            lambda.1.smry <- unlist(list(lambda.hat.1, t.0.hat.1, m.ln.like.1))
            lambda.2.smry <- unlist(list(lambda.hat.2, t.0.hat.2, m.ln.like.2))
            lambda.smry <- rbind(lambda.1.smry, lambda.2.smry)
            colnames(lambda.smry) <- c("lambda", "t.0", "ln.L")
        
            setwd(paste(lambdat0.path, "Pairwise_tests", sep="/"))
            write.table(lambda.smry, file=paste(mutant.1, ".", mutant.2, ".lambda.LRT.smry.txt", sep=""), sep="\t", col.names=T, row.names=F)
        
        
            null.lam.table <- as.data.frame(matrix(nrow=length(lambda.v), ncol=5))
            rownames(null.lam.table) <- lambda.v
            colnames(null.lam.table) <- c("mut1.t0", "mut1.lnL", "mut2.t0", "mut2.lnL", "lnL.null")
            for (lam.i in 1:length(lambda.v)){
                m.1 <- max(t.0.by.lam.1[,lam.i])
                r.1 <- which(t.0.by.lam.1[,lam.i]==m.1)
                null.lam.table$mut1.t0[lam.i] <- t.0.v[r.1]
                null.lam.table$mut1.lnL[lam.i] <- m.1
            
                m.2 <- max(t.0.by.lam.2[,lam.i])
                r.2 <- which(t.0.by.lam.2[,lam.i]==m.2)
                null.lam.table$mut2.t0[lam.i] <- t.0.v[r.2]
                null.lam.table$mut2.lnL[lam.i] <- m.2
                
                null.lam.table$lnL.null[lam.i] <- m.1 + m.2         
            }
        
            m.3 <- max(null.lam.table$lnL.null)
            r.3 <- which(null.lam.table$lnL.null==m.3)
            max.lambda <- lambda.v[r.3]
            max.lnL.null <- m.3
            t.0.1.null <- null.lam.table$mut1.t0[r.3]
            t.0.2.null <- null.lam.table$mut2.t0[r.3]
            
            max.lnL.alt <- m.ln.like.1 + m.ln.like.2
            LR.obs <- max.lnL.alt - max.lnL.null
            
            
            
        
            # === Now we need the distribution of LR.obs under the null ===

            LR.boots <- rep(NA, n.boot.reps)
        
            for (boot.i in 1:n.boot.reps){
                print(paste("bootstrap rep.i=", boot.i))
                sim.data.null.1 <- sim.dataset(max.lambda, alpha, t.0.1.null, all.data.1)
                sim.data.null.2 <- sim.dataset(max.lambda, alpha, t.0.2.null, all.data.2)
                    
                lambda.results.1 <- find.lambda.hat(sim.data.null.1, t.0.v, lambda.v)
                lambda.hat.1 <- lambda.results.1$lambda.hat
                t.0.hat.1 <- lambda.results.1$t.0.hat
                m.ln.like.1 <- lambda.results.1$max.log.likelihood
                t.0.by.lam.1 <- lambda.results.1$t.0.by.lam
            
                lambda.results.2 <- find.lambda.hat(sim.data.null.2, t.0.v, lambda.v)
                lambda.hat.2 <- lambda.results.2$lambda.hat
                t.0.hat.2 <- lambda.results.2$t.0.hat
                m.ln.like.2 <- lambda.results.2$max.log.likelihood
                t.0.by.lam.2 <- lambda.results.2$t.0.by.lam
    
                boot.lam.table <- as.data.frame(matrix(nrow=length(lambda.v), ncol=5))
                rownames(boot.lam.table) <- lambda.v
                colnames(boot.lam.table) <- c("mut1.t0", "mut1.lnL", "mut2.t0", "mut2.lnL", "lnL.null")
                for (lam.i in 1:length(lambda.v)){
                    m.1 <- max(t.0.by.lam.1[,lam.i])
                    r.1 <- which(t.0.by.lam.1[,lam.i]==m.1)
                    boot.lam.table$mut1.t0[lam.i] <- t.0.v[r.1]
                    boot.lam.table$mut1.lnL[lam.i] <- m.1
                
                    m.2 <- max(t.0.by.lam.2[,lam.i])
                    r.2 <- which(t.0.by.lam.2[,lam.i]==m.2)
                    boot.lam.table$mut2.t0[lam.i] <- t.0.v[r.2]
                    boot.lam.table$mut2.lnL[lam.i] <- m.2
                
                    boot.lam.table$lnL.null[lam.i] <- m.1 + m.2         
                }
            
                m.32 <- max(boot.lam.table$lnL.null)
                r.32 <- which(boot.lam.table$lnL.null==m.32)
                boot.lambda <- lambda.v[r.32]
                boot.lnL.null <- m.32
                t.0.1.boot <- null.lam.table$mut1.t0[r.32]
                t.0.2.boot <- null.lam.table$mut2.t0[r.32]
            
                boot.lnL.alt <- m.ln.like.1 + m.ln.like.2
                LR.boots[boot.i] <- boot.lnL.alt - boot.lnL.null
            
            }  #next boot.i
            
            setwd(paste(lambdat0.path, "Pairwise_tests", sep="/"))
            write.table(LR.obs, file=paste(mutant.1, ".", mutant.2, ".lambda.LRT.LR.obs(2).txt", sep=""), sep="\t", col.names=T, row.names=F)
            write.table(LR.boots, file=paste(mutant.1, ".", mutant.2, ".lambda.LRT.LR.boots(2).txt", sep=""), sep="\t", col.names=T, row.names=F)
        
            p.vals.lam[mut.i, mut.j] <- length(which(LR.boots > LR.obs))/n.boot.reps
        }   # next mut.j
    }    # next mut.i
      
  } else{  # end if (run.pairwise.lambda.tests == TRUE)...
    print("Note: not running test for differences in lambda. Instead reading results from file.") 
  }
```

```
## [1] "Note: not running test for differences in lambda. Instead reading results from file."
```

```
  setwd(paste(lambdat0.path, "Pairwise_tests", sep="/"))    
  p.vals.lam <- read.table(file="lambda.LRT.pvals.lam(2).txt", sep="\t", header=TRUE)
  print(p.vals.lam)
```

```
##       WT m.319 m.321 m.323 m.324   m.5   m.6  m.4B
## WT    NA  0.01 0.000 0.028 0.018 0.006 0.322 0.506
## m.319 NA    NA 0.404 0.888 0.870 0.844 0.082 0.028
## m.321 NA    NA    NA 0.260 0.314 0.464 0.010 0.000
## m.323 NA    NA    NA    NA 0.836 0.702 0.136 0.092
## m.324 NA    NA    NA    NA    NA 0.752 0.078 0.042
## m.5   NA    NA    NA    NA    NA    NA 0.068 0.022
## m.6   NA    NA    NA    NA    NA    NA    NA 0.780
## m.4B  NA    NA    NA    NA    NA    NA    NA    NA
```

Table shows the p-values associated with the null that \(\lambda\) is the same between pair (while \(t\_0\) is free to vary).

### Test for differences in \(t\_0\) b/t pairs of clones

```
    # ==== Now the same analysis, but hold t.0 fixed between two mutants under null and allow lambda to vary freely. ===
    # === Answers the question: is t.0 signficantly different between two mutants                                     ===
  
    if (run.pairwise.t0.tests == TRUE){
      n.boot.reps  <- n.pairwist.lambdat0.boots     
      p.vals.t0 <- as.data.frame(matrix(nrow=n.muts, ncol=n.muts))
      colnames(p.vals.t0) <- mutant.v
      rownames(p.vals.t0) <- mutant.v
      
      for (mut.i in 1:(n.muts-1)){
          for (mut.j in (mut.i+1):n.muts){
            print(paste("===== mut.i=", mut.i, "   mut.j=", mut.j, " ====="))

            preped.data.1 <- prep.data(data.set.1)
            all.data.1 <- preped.data.1$all.data
            dates.1 <- preped.data.1$dates
            beta.by.day.1 <- preped.data.1$beta.by.day
            burst.wells.1 <- preped.data.1$burst.wells
            burst.times.1 <- preped.data.1$burst.times
            max.burst.1 <- preped.data.1$max.burst 
            
            preped.data.2 <- prep.data(data.set.2)
            all.data.2 <- preped.data.2$all.data
            dates.2 <- preped.data.2$dates
            beta.by.day.2 <- preped.data.2$beta.by.day
            burst.wells.2 <- preped.data.2$burst.wells
            burst.times.2 <- preped.data.2$burst.times
            max.burst.2 <- preped.data.2$max.burst 
            
            print("Analyzing mut.i")
            
            lambda.results.1 <- find.lambda.hat(all.data.1, t.0.v, lambda.v)
            lambda.hat.1 <- lambda.results.1$lambda.hat
            t.0.hat.1 <- lambda.results.1$t.0.hat
            m.ln.like.1 <- lambda.results.1$max.log.likelihood
            lambda.1.CI <- lambda.results.1$lambda.CI
            t.0.1.CI <- lambda.results.1$t.0.CI
            t.0.by.lam.1 <- lambda.results.1$t.0.by.lam
            
            print("Analyzing mut.j")
        
            lambda.results.2 <- find.lambda.hat(all.data.2, t.0.v, lambda.v)
            lambda.hat.2 <- lambda.results.2$lambda.hat
            t.0.hat.2 <- lambda.results.2$t.0.hat
            m.ln.like.2 <- lambda.results.2$max.log.likelihood
            lambda.2.CI <- lambda.results.2$lambda.CI
            t.0.2.CI <- lambda.results.2$t.0.CI
            t.0.by.lam.2 <- lambda.results.2$t.0.by.lam
            
            lambda.1.smry <- unlist(list(lambda.hat.1, t.0.hat.1, m.ln.like.1))
            lambda.2.smry <- unlist(list(lambda.hat.2, t.0.hat.2, m.ln.like.2))
            lambda.smry <- rbind(lambda.1.smry, lambda.2.smry)
            colnames(lambda.smry) <- c("lambda", "t.0", "ln.L")
            
            setwd(paste(lambdat0.path, "Pairwise_tests", sep="/"))
            write.table(lambda.smry, file=paste(mutant.1, ".", mutant.2, ".t0.LRT.smry.txt", sep=""), sep="\t", col.names=T, row.names=F)
            
            
            null.t0.table <- as.data.frame(matrix(nrow=length(t.0.v), ncol=5))
            rownames(null.t0.table) <- t.0.v
            colnames(null.t0.table) <- c("mut1.lam", "mut1.lnL", "mut2.lam", "mut2.lnL", "lnL.null")
            for (t0.i in 1:length(t.0.v)){
                m.1 <- max(t.0.by.lam.1[t0.i,])
                r.1 <- which(t.0.by.lam.1[t0.i,]==m.1)
                null.t0.table$mut1.lam[t0.i] <- lambda.v[r.1]
                null.t0.table$mut1.lnL[t0.i] <- m.1
                
                m.2 <- max(t.0.by.lam.2[t0.i,])
                r.2 <- which(t.0.by.lam.2[t0.i,]==m.2)
                null.t0.table$mut2.lam[t0.i] <- lambda.v[r.2]
                null.t0.table$mut2.lnL[t0.i] <- m.2
                
                null.t0.table$lnL.null[t0.i] <- m.1 + m.2           
            }
            
            m.3 <- max(null.t0.table$lnL.null)
            r.3 <- which(null.t0.table$lnL.null==m.3)
            max.t0 <- t.0.v[r.3]
            max.lnL.null <- m.3
            lam.1.null <- null.t0.table$mut1.lam[r.3]
            lam.2.null <- null.t0.table$mut2.lam[r.3]
            
            max.lnL.alt <- m.ln.like.1 + m.ln.like.2
            LR.obs <- max.lnL.alt - max.lnL.null
            
            
            
        
            # === Now we need the distribution of LR.obs under the null ===

            LR.boots <- rep(NA, n.boot.reps)
        
            for (boot.i in 1:n.boot.reps){
                print(paste("bootstrap rep.i=", boot.i))
                sim.data.null.1 <- sim.dataset(lam.1.null, alpha, max.t0, all.data.1)
                sim.data.null.2 <- sim.dataset(lam.2.null, alpha, max.t0, all.data.2)
                    
                lambda.results.1 <- find.lambda.hat(sim.data.null.1, t.0.v, lambda.v)
                lambda.hat.1 <- lambda.results.1$lambda.hat
                t.0.hat.1 <- lambda.results.1$t.0.hat
                m.ln.like.1 <- lambda.results.1$max.log.likelihood
                t.0.by.lam.1 <- lambda.results.1$t.0.by.lam
            
                lambda.results.2 <- find.lambda.hat(sim.data.null.2, t.0.v, lambda.v)
                lambda.hat.2 <- lambda.results.2$lambda.hat
                t.0.hat.2 <- lambda.results.2$t.0.hat
                m.ln.like.2 <- lambda.results.2$max.log.likelihood
                t.0.by.lam.2 <- lambda.results.2$t.0.by.lam
    
                boot.t0.table <- as.data.frame(matrix(nrow=length(t.0.v), ncol=5))
                rownames(boot.t0.table) <- t.0.v
                colnames(boot.t0.table) <- c("mut1.lam", "mut1.lnL", "mut2.lam", "mut2.lnL", "lnL.null")
                for (t0.i in 1:length(t.0.v)){
                    m.1 <- max(t.0.by.lam.1[t0.i,])
                    r.1 <- which(t.0.by.lam.1[t0.i,]==m.1)
                    boot.t0.table$mut1.lam[t0.i] <- lambda.v[r.1]
                    boot.t0.table$mut1.lnL[t0.i] <- m.1
                
                    m.2 <- max(t.0.by.lam.2[t0.i,])
                    r.2 <- which(t.0.by.lam.2[t0.i,]==m.2)
                    boot.t0.table$mut2.lam[t0.i] <- lambda.v[r.2]
                    boot.t0.table$mut2.lnL[t0.i] <- m.2
                
                    boot.t0.table$lnL.null[t0.i] <- m.1 + m.2           
                }
            
                m.32 <- max(boot.t0.table$lnL.null)
                r.32 <- which(boot.t0.table$lnL.null==m.32)
                boot.t0 <- t.0.v[r.32]
                boot.lnL.null <- m.32
                lam.1.boot <- null.t0.table$mut1.lam[r.32]
                lam.2.boot <- null.t0.table$mut2.lam[r.32]
            
                boot.lnL.alt <- m.ln.like.1 + m.ln.like.2
                LR.boots[boot.i] <- boot.lnL.alt - boot.lnL.null
            
            }  #next boot.i
            
            setwd(paste(lambdat0.path, "Pairwise_tests", sep="/"))
            write.table(LR.obs, file=paste(mutant.1, ".", mutant.2, ".t0.LRT.LR.obs(2).txt", sep=""), sep="\t", col.names=T, row.names=F)
            write.table(LR.boots, file=paste(mutant.1, ".", mutant.2, ".t0.LRT.LR.boots(2).txt", sep=""), sep="\t", col.names=T, row.names=F)
            
            p.vals.t0[mut.i, mut.j] <- length(which(LR.boots > LR.obs))/n.boot.reps
        }   # next mut.j
    }    # next mut.i
    
    write.table(p.vals.t0, file="t0.LRT.pvals.lam(2).txt", sep="\t", col.names=T, row.names=T)
    
  } else{ # end if (run.pairwise.t0.tests == TRUE)...
    print("Note: not running test for differences in t0. Instead reading results from file.")  
  }
```

```
## [1] "Note: not running test for differences in t0. Instead reading results from file."
```

```
  setwd(paste(lambdat0.path, "Pairwise_tests", sep="/"))    
  p.vals.t0 <- read.table(file="t0.LRT.pvals.lam(2).txt", sep="\t", header=TRUE)
  print(p.vals.t0)
```

```
##       WT m.319 m.321 m.323 m.324   m.5   m.6  m.4B
## WT    NA 0.478 0.000 0.050 0.084 0.210 0.038 0.552
## m.319 NA    NA 0.006 0.132 0.334 0.478 0.002 0.910
## m.321 NA    NA    NA 0.246 0.044 0.014 0.000 0.004
## m.323 NA    NA    NA    NA 0.486 0.364 0.002 0.142
## m.324 NA    NA    NA    NA    NA 0.698 0.000 0.276
## m.5   NA    NA    NA    NA    NA    NA 0.004 0.478
## m.6   NA    NA    NA    NA    NA    NA    NA 0.006
## m.4B  NA    NA    NA    NA    NA    NA    NA    NA
```

Table shows the p-values associated with the null hypothesis that \(t\_0\) is the same between clones (while \(\lambda\) is free to vary).

### Estimating Burst Size as Function of Time

```
  #  The model for this is simple: Burst size = alpha*time + mu + epsilon where 
  #     alpha = slope
  #     mu = intercept (burst size at time t.0), and 
  #       epsilon = error (assumed to be normal mean 0, variance sigma squared that doesn't change in time)
  #       It turns out to be complicated in practice b/c we don't see burst size, rather we see the sum of burst sizes from an unknown # of phage.
  #       We don't see time of lysis either. Rather, we only know the time we sampled the well as opposed to when the burst event occurred.
  #       We'll be using the EM algorithm to deal with these latent (unknown) variables.
  #     EM or em in the code refers to the EM algorithm, or "expectation-maximization."
  #     The EM idea is when you don't know a latent variable that you need to know in order to calculate the likelihood,
  #     replace it with its expected value. But you can't calculate these expectations without knowing other parameters that you
  #     are estimating. So the process is iterative: guess parameters, calculate expected values, use expected values to estimate parameters (via maximum likelihood).
  #     Given these parameter estimates, calculate expected values, etc. etc. etc. until things converge. That's EM.
  #     It is helpful to define some things.
  #     N = the # of phage in a well that have burst by time t where 
  #     t = the time we sample a well
  #     T = the time that lysis actually occured (as opposed to when we sampled the well)
  #     E[N] E[1.N] are the exptected values of N and 1/N. We'll want these for each burst well.
  #     X = when a well is sampled at time t, N phage have lysed their hosts. Add up all the times it took those N phage to lyse their hosts, that is X.
  #     E[X] = the expected value of X. Like E[N] we'll want to know this expectation for each burst well.
  #     Well also need E[X^2] and other related expectations.
  #     A note about time:  once we estimate t.0, redefine all timepoints relative to this one. Thus all sample times for burst wells are measured
  #     as time since t.0. This makes the math a lot easier.
  #     Note, we'll need to find E[X] and E[N]. This would be easier if they were independent, but they are not. The larger N is, the larger X tends to be.
  #     Thus one thing we'll need is the joint probability distribution of N and X. We'll get this by simulation. The good news is that we only need to simulate 
  #     this once for each day (each beta value). There are some other things that we need to do before running the EM algorithm described above.
  
  #     BASIC IDEA: (1) Simulate a distribution (joint P(X and N)) we need, but that only needs to be simulated once. Do this at the beginning. 
  #             (2) Define # of iterations and dimension some matrices we'll need.
  #             (3) Other time related preliminaries: shift times give t.0. Determine E[t] given T for each well that shows a lysis event. Find probabilty of burst for each timept after t.0. 
  #             (4) Ballparking: Get rough but reasonable initial parameter estimates. Uses some rough, ad hoc methods to get reasonable starting places.
  #             (5) Given parameter estimates, get intial values for the expectations such as E[N] and E[X], etc.
  #             (6) Given these expected values (from 5), estimate parameter values (alpha, mu, sigma).
  #             (7) Run the EM Algorithm for a prespecified number of steps. 
  #             (8) Generate some plots


  
  if (run.burstsize.regression ==TRUE){
    
    prob.N.X.list <- vector(mode="list", length=n.muts)
    joint.time.list <- vector(mode="list", length=n.muts)
    x.scale.list <- vector(mode="list", length=n.muts)
    
    # ====== STAGE I. get the joint N, X distribution (prob.N.X.list)=======
    # ====== This does that for each mutant, each assay (beta) =======
  
    for (mut.i in 1:n.muts){  
        
        print(paste("mut.i=", mut.i))
        mutant <- mutant.v[mut.i]
        new.tiger.data <- data[[mut.i]]
    
        all.data <- matrix(nrow=1, ncol=6)              # new data is matrix where all observations are concatenated--1 row per well/plate
        colnames(all.data) <- c("burst", "time", "beta", "date", "batch", "dataset")   # burst=burst size...batch is identifier in case > 1 datasets are done on same day using different stock
        all.data <- as.data.frame(all.data)
    
        dates <- colnames(new.tiger.data)               # get assay dates from column names
        dates <- dates[-1]                             # remove 1st column header (time)
        n.days <- length(dates)                        # number of days
        time.v <- new.tiger.data[,1]
        beta.by.day <- matrix(nrow=1, ncol=n.days)
        colnames(beta.by.day) <- dates
        
        
        
        setwd(lambdat0.path)
        mut.pt.est <- read.table(file=paste(mutant, ".lambda.t0.est.txt", sep=""), header=T, sep="\t")
        t.0.hat <- mut.pt.est$t.0.hat
        lambda.hat <- mut.pt.est$lambda.hat
        alpha.hat <- 1
        
        # Estimate beta for each day (i.e. each column)
        for (day.i in 1:n.days){
            day.v.raw <- new.tiger.data[,day.i+1]   # b/c 1st column is time, data is right-shifted 1 column
            not.NA <- which(is.na(day.v.raw)==FALSE)
            day.v <- day.v.raw[not.NA]
            beta.by.day[day.i] <- est.beta.day.NEW(day.v)$beta.hat
            }
            
            
            #   Put data with beta estimtes into all.data
            for (day.i in 1:n.days){
                for (time.i in 1:length(time.v)){
                    if (is.na(new.tiger.data[time.i, day.i+1])==FALSE){   # want to skip NA cells (no data)
                        data.row <- rep(NA, length(all.data[1,]))
                        data.row[1] <- new.tiger.data[time.i, day.i + 1]
                        data.row[2] <- new.tiger.data[time.i, 1]            
                        data.row[3]<- beta.by.day[day.i]
                        data.row[4] <- dates[day.i]
                        all.data <- rbind(all.data, data.row)
                        }
                    }
                }
    
            #  Remove first row of all.data (only there in first place to allow concatenation onto)
              all.data <- all.data[-1,]
              burst.wells <- which(abs(as.numeric(all.data$burst))>burst.cutoff)   # note use of absolute value so overgrown plates are included as burst wells
              burst.times <- as.numeric(all.data$time[burst.wells])
              max.burst <- max(burst.times)
    
    
        # ======== (2) SETTINGS & DIMENSIONING...=========
            #n.em.iterations <- 500
            all.data.h <- as.data.frame(all.data)
            real.estimates.EM <- matrix(nrow=n.em.iterations, ncol=3)   # , 1=slope, 2=intercept, 3=sigma^2
            real.estimates.EM.og <- matrix(nrow=n.em.iterations, ncol=3)   # , 1=slope, 2=intercept, 3=sigma^2
            colnames(real.estimates.EM) <- c("alpha(slope)", "mu(intercept)", "sigma")
            burst.wells <- which(as.numeric(all.data.h$burst)>burst.cutoff)
            bursts <- as.numeric(all.data.h$burst[burst.wells]) 
            overgrown.wells <- which(as.numeric(all.data.h$burst) < -100)   #not defining how to recognize overgrown cells right here.  
            overgrown.values <- as.numeric(all.data.h$burst[overgrown.wells])
            overgrown.method <- "exclude"
        
            E.N.real <- matrix(nrow=length(burst.wells), ncol=n.em.iterations)
            E.X.real <- matrix(nrow=length(burst.wells), ncol=n.em.iterations)
            E.X2.N.real <- matrix(nrow=length(burst.wells), ncol=n.em.iterations)
            E.XC.N.real <- matrix(nrow=length(burst.wells), ncol=n.em.iterations)
            E.1.N.real <- matrix(nrow=length(burst.wells), ncol=n.em.iterations)
            E.X.N.real <- matrix(nrow=length(burst.wells), ncol=n.em.iterations)
            
        
        
        # ===== (3) OTHER PRELIMINARIES =====
            # Note that E.burst.time, E[t] and the expected square of the time, E[t^2], only have meaning for wells that have burst.
            t <- seq(0, max(time.v)-t.0.hat, by=0.25)
            t.adj <- t + t.0.hat
            E.burst.time <- function(t) ((lambda.hat-t*exp(-t/lambda.hat))-lambda.hat*exp(-t/lambda.hat))/(1-exp(-t/lambda.hat))
            E.burst.time.2 <- function(t) (2*lambda.hat^2 - t^2*exp(-t/lambda.hat) - 2*t*lambda.hat*exp(-t/lambda.hat) - 2*lambda.hat^2*exp(-t/lambda.hat))/(1-exp(-t/lambda.hat))  
            E.burst.t <- rep(NA, length(all.data$time))
            E.burst.t.2 <- rep(NA, length(all.data$time))
            shifted.times <- as.numeric(all.data$time) - t.0.hat
            for (plate.i in 1:length(all.data[,1])){
                if (abs(as.numeric(all.data[plate.i,1])) > burst.cutoff){
                    E.burst.t[plate.i] <- E.burst.time(shifted.times[plate.i])
                    E.burst.t.2[plate.i] <- E.burst.time.2(shifted.times[plate.i])
                    }
                }
            all.data <- cbind(all.data, shifted.times, E.burst.t, E.burst.t.2)
            
            #prob.burst.at.t <- function(t) (1/lambda.hat)*exp(-t/lambda.hat)
            
            s.h <- seq(0, max.burst + 1, by=0.25)               # a vector of timepoints from zero to the max sampled.
            w.t.v <- generate.w.t.v(lambda.hat, t.0.hat, alpha.hat, s.h)        # gets the probability of having lysed by each time in s.h
            day.v <- unique(all.data.h$date)
        
            all.data.h <- all.data
            all.data.h$burst[overgrown.wells] <- NA
            #all.data.h <- get.E.N.for.dataset(all.data.h, int.i, slope.i, sig.hat.i)  # for initial values of intercept, slope, and sigma sq
        # ===============================
        
        # ===== (1) CREATE JOINT N.t by X.t DISTRIBUTION ======
            # TAKES A FEW MINUTES PER DAY (PER BETA VALUE). 
            # But only need to run once.
            joint.time.v <- unique(all.data$shifted.times)
            joint.time.v <- joint.time.v[which(joint.time.v >= 0)]
            x.scale <- c(seq(0.1,0.9, by=0.1), seq(1,5,by=0.25), seq(6,100, by=1))
            prob.N.X <- get.N.t.X.t.joint(beta.by.day, joint.time.v, x.scale, lambda.hat, 100000)  # get back vast array of probabilities w/ these dims:  
                                                                               # 1st = value of X (sum.T); 2nd = N.t; 3rd = each time point in joint.time.v(t); 4th = each day (value of beta)
                                                                               # 5th value = number of observations to collect (use 100000 for real analysis)
            joint.time.list[[mut.i]] <- joint.time.v
            x.scale.list[[mut.i]] <- x.scale
            prob.N.X.list[[mut.i]] <- prob.N.X
    }  # next mut.i
    
    
    
    
    
    # ======== STAGE II.  ACTUALLY DO THE REGRESSION ========================
    #====== OK, now use prob.N.X.list to regression burst size against time ==
    
    for (mut.i in 1:n.muts){
      print(paste("mut.i=", mut.i))
        mutant <- mutant.v[mut.i]
        new.tiger.data <- data[[mut.i]]
    
        all.data <- matrix(nrow=1, ncol=6)              # new data is matrix where all observations are concatenated--1 row per well/plate
        colnames(all.data) <- c("burst", "time", "beta", "date", "batch", "dataset")   # burst=burst size...batch is identifier in case > 1 datasets are done on same day using different stock
        all.data <- as.data.frame(all.data)
    
        dates <- colnames(new.tiger.data)               # get assay dates from column names
        dates <- dates[-1]                             # remove 1st column header (time)
        n.days <- length(dates)                        # number of days
        time.v <- new.tiger.data[,1]
        beta.by.day <- matrix(nrow=1, ncol=n.days)
        colnames(beta.by.day) <- dates
        
        setwd(lambdat0.path)
        mut.pt.est <- read.table(file=paste(mutant, ".lambda.t0.est.txt", sep=""), header=T, sep="\t")
        t.0.hat <- mut.pt.est$t.0.hat
        lambda.hat <- mut.pt.est$lambda.hat
        alpha.hat <- 1
        
        prob.N.X <- prob.N.X.list[[mut.i]]
        joint.time.v <- joint.time.list[[mut.i]]
        x.scale <- x.scale.list[[mut.i]]
        
        
        # Estimate beta for each day (i.e. each column)
        for (day.i in 1:n.days){
            day.v.raw <- new.tiger.data[,day.i+1]   # b/c 1st column is time, data is right-shifted 1 column
            not.NA <- which(is.na(day.v.raw)==FALSE)
            day.v <- day.v.raw[not.NA]
            beta.by.day[day.i] <- est.beta.day.NEW(day.v)$beta.hat
        }
            
            
            #   Put data with beta estimtes into all.data
            for (day.i in 1:n.days){
                for (time.i in 1:length(time.v)){
                    if (is.na(new.tiger.data[time.i, day.i+1])==FALSE){   # want to skip NA cells (no data)
                        data.row <- rep(NA, length(all.data[1,]))
                        data.row[1] <- new.tiger.data[time.i, day.i + 1]
                        data.row[2] <- new.tiger.data[time.i, 1]            
                        data.row[3]<- beta.by.day[day.i]
                        data.row[4] <- dates[day.i]
                        all.data <- rbind(all.data, data.row)
                        }
                    }
                }
    
            #  Remove first row of all.data (only there in first place to allow concatenation onto)
            all.data <- all.data[-1,]
            burst.wells <- which(abs(as.numeric(all.data$burst))>burst.cutoff)   # note use of absolute value so overgrown plates are included as burst wells
            burst.times <- as.numeric(all.data$time[burst.wells])
            max.burst <- max(burst.times)

            
            
            # ======== (2) SETTINGS & DIMENSIONING...=========
            #n.em.iterations <- 500
            all.data.h <- as.data.frame(all.data)
            real.estimates.EM <- matrix(nrow=n.em.iterations, ncol=3)   # , 1=slope, 2=intercept, 3=sigma^2
            real.estimates.EM.og <- matrix(nrow=n.em.iterations, ncol=3)   # , 1=slope, 2=intercept, 3=sigma^2
            colnames(real.estimates.EM) <- c("alpha(slope)", "mu(intercept)", "sigma")
            burst.wells <- which(as.numeric(all.data.h$burst)>burst.cutoff)
            bursts <- as.numeric(all.data.h$burst[burst.wells]) 
            overgrown.wells <- which(as.numeric(all.data.h$burst) < -100)   #not defining how to recognize overgrown cells right here.  
            overgrown.values <- as.numeric(all.data.h$burst[overgrown.wells])
            overgrown.method <- "exclude"
        
            E.N.real <- matrix(nrow=length(burst.wells), ncol=n.em.iterations)
            E.X.real <- matrix(nrow=length(burst.wells), ncol=n.em.iterations)
            E.X2.N.real <- matrix(nrow=length(burst.wells), ncol=n.em.iterations)
            E.XC.N.real <- matrix(nrow=length(burst.wells), ncol=n.em.iterations)
            E.1.N.real <- matrix(nrow=length(burst.wells), ncol=n.em.iterations)
            E.X.N.real <- matrix(nrow=length(burst.wells), ncol=n.em.iterations)
            
        
        
        # ===== (3) OTHER PRELIMINARIES =====
            # Note that E.burst.time, E[t] and the expected square of the time, E[t^2], only have meaning for wells that have burst.
            t <- seq(0, max(time.v)-t.0.hat, by=0.25)
            t.adj <- t + t.0.hat
            E.burst.time <- function(t) ((lambda.hat-t*exp(-t/lambda.hat))-lambda.hat*exp(-t/lambda.hat))/(1-exp(-t/lambda.hat))
            E.burst.time.2 <- function(t) (2*lambda.hat^2 - t^2*exp(-t/lambda.hat) - 2*t*lambda.hat*exp(-t/lambda.hat) - 2*lambda.hat^2*exp(-t/lambda.hat))/(1-exp(-t/lambda.hat))  
            E.burst.t <- rep(NA, length(all.data$time))
            E.burst.t.2 <- rep(NA, length(all.data$time))
            shifted.times <- as.numeric(all.data$time) - t.0.hat
            for (plate.i in 1:length(all.data[,1])){
                if (abs(as.numeric(all.data[plate.i,1])) > burst.cutoff){
                    E.burst.t[plate.i] <- E.burst.time(shifted.times[plate.i])
                    E.burst.t.2[plate.i] <- E.burst.time.2(shifted.times[plate.i])
                    }
                }
            all.data <- cbind(all.data, shifted.times, E.burst.t, E.burst.t.2)
            
            #prob.burst.at.t <- function(t) (1/lambda.hat)*exp(-t/lambda.hat)
            
            s.h <- seq(0, max.burst + 1, by=0.25)               # a vector of timepoints from zero to the max sampled.
            w.t.v <- generate.w.t.v(lambda.hat, t.0.hat, alpha.hat, s.h)        # gets the probability of having lysed by each time in s.h
            day.v <- unique(all.data.h$date)
        
            all.data.h <- all.data
            all.data.h$burst[overgrown.wells] <- NA
            #all.data.h <- get.E.N.for.dataset(all.data.h, int.i, slope.i, sig.hat.i)  # for initial values of intercept, slope, and sigma sq
        # ===============================
            
            # ====== (4) BALLPARKING: Get intial estimates for parameters ========
            # Uses rough estimates from the early bursts when time
            # has had a minimal effect on burst size.
            if (length(day.v)>1){   
                early.t <- 3.5   # just says that bursts before t.0+early.t will be considered early burts for use in estimting mu initial.
                mode.by.time.list <- mode.by.time.estimation(all.data.h)
                mode.regression <- mode.by.time.regression(mode.by.time.list)
                intercept.i <-  mode.regression$mode.lm.var.wt$coefficients[1]
                slope.i <-  mode.regression$mode.lm.var.wt$coefficients[2]
                early.wells <- which(all.data.h$shifted.times<=early.t) 
                early.burst.wells <- intersect(early.wells, burst.wells)
                e.counts <- as.numeric(all.data.h$burst[early.burst.wells])
                d.e <- density(e.counts)
                mu.e <- d.e$x[which.max(d.e$y)]
                sigma.i <- est.sig.half.normal(mu.e, e.counts)  
            } else{             # In the event you have just 1 day's data, input the starting parameter values.
                slope.i <- 5
                intercept.i <- 40
                sigma.i <- 20
            }
            #========================================================================
            
            # ==== (5) Get initial values for E.N, E.X... ================
            for (well.i in 1:length(burst.wells)){
                well <- burst.wells[well.i]
                burst <- bursts[well.i]
                day.i <- which(all.data.h$date[well]==day.v)
                time.i.shifted <- which(all.data.h$shifted.times[well]==joint.time.v)
                E.N.X.etc <- find.expected.X.N.etc.by.datapoint(prob.N.X, burst, slope.i, intercept.i, sigma.i, day.i, time.i.shifted, x.scale)
                        
                E.N.real[well.i, 1] <- E.N.X.etc$E.N.giv.C 
                E.X.real[well.i, 1] <- E.N.X.etc$E.X.giv.C 
                E.X2.N.real[well.i, 1] <- E.N.X.etc$E.X2.N.giv.C 
                E.XC.N.real[well.i, 1] <- E.N.X.etc$E.XC.N.giv.C
                E.1.N.real[well.i, 1] <- E.N.X.etc$E.1.N.giv.C
                E.X.N.real[well.i, 1] <- E.N.X.etc$E.X.N.giv.C
                }   
            # ========================================================
            
            
            # ===== (6) UPDATE ESTIMATES OF ALPHA, MU, AND SIGMA ========
            real.estimates.EM[1, ] <- estimate.alpha.mu.sigma(bursts, E.N.real[,1], E.X.real[,1], E.X2.N.real[,1], E.XC.N.real[,1], 
                                                              E.1.N.real[,1], E.X.N.real[,1] , all.data.h$E.burst.t[burst.wells], 
                                                              all.data.h$E.burst.t.2[burst.wells])[1:3]
            
            # ===== (7) NOW ITERATE EM-ALGORITHM ========================
            for (iteration in 2:n.em.iterations){
                print(paste("iteration=", iteration))
                for (well.i in 1:length(burst.wells)){
                        burst <- bursts[well.i]
                        if (burst > burst.cutoff){
                            well <- burst.wells[well.i]
                            day.i <- which(all.data.h$date[well]==day.v)
                            time.i <- which(as.numeric(all.data.h$time[well])==s.h)
                            time.i.shifted <- which(all.data.h$shifted.times[well]==joint.time.v)
                            shifted.time <- all.data.h$E.burst.t[well]
                            intercept <- real.estimates.EM[iteration-1, 2]
                            slope <- real.estimates.EM[iteration-1, 1]
                            sigma <- real.estimates.EM[iteration-1, 3]
                            mu.this.well <- intercept + slope*shifted.time
                        
                            E.N.X.etc <- find.expected.X.N.etc.by.datapoint(prob.N.X, burst, slope, intercept, sigma, day.i, time.i.shifted, x.scale)
                            E.N.real[well.i, iteration] <- E.N.X.etc$E.N.giv.C 
                            E.X.real[well.i, iteration] <- E.N.X.etc$E.X.giv.C 
                            E.X2.N.real[well.i, iteration] <- E.N.X.etc$E.X2.N.giv.C 
                            E.XC.N.real[well.i, iteration] <- E.N.X.etc$E.XC.N.giv.C
                            E.1.N.real[well.i, iteration] <- E.N.X.etc$E.1.N.giv.C
                            E.X.N.real[well.i, iteration] <- E.N.X.etc$E.X.N.giv.C
                        }  # end if burst>burst.cutoff 
                    }  # next well.i
                        
                        real.estimates.EM[iteration, ] <- estimate.alpha.mu.sigma(bursts, E.N.real[,iteration], E.X.real[,iteration], E.X2.N.real[,iteration], E.XC.N.real[,iteration], E.1.N.real[,iteration], E.X.N.real[,iteration], all.data.h$E.burst.t[burst.wells], all.data.h$E.burst.t.2[burst.wells])[1:3]
                } #next iteration
            
            alph.v <- real.estimates.EM[,1]  # slope
                mu.v <- real.estimates.EM[,2]    # intercept
                sig.v <- real.estimates.EM[,3]   # variability
                slope.i <- alph.v[n.em.iterations]
                slope.b <- slope.i                  # i = initial (for initial round w/o overgrowns; b=2nd round. Will be updated w/ each iteration)
                intercept.i <- mu.v[n.em.iterations]    
                intercept.b <- intercept.i
                sigma.i <- sig.v[n.em.iterations]
                sigma.b <- sigma.i
                burst.size.time.pt.ests <- cbind(slope=slope.i, intercpet=intercept.i, sigma=sigma.i)
                
                setwd(bustsize.path)
                write.table(alph.v, file=paste(mutant, ".slope.v.txt", sep=""), append=FALSE, sep="\t", col.names=TRUE, row.names=FALSE)
                write.table(mu.v, file=paste(mutant, ".intercept.v.txt", sep=""), append=FALSE, sep="\t", col.names=TRUE, row.names=FALSE)
                write.table(sig.v, file=paste(mutant, ".sigma.v.txt", sep=""), append=FALSE, sep="\t", col.names=TRUE, row.names=FALSE)
                write.table(burst.size.time.pt.ests, file=paste(mutant, ".slope.int.sig.pt.ests.txt", sep=""), append=FALSE, sep="\t", col.names=TRUE, row.names=FALSE)
                write.table(x=E.X.real, file=paste(mutant, ".E.X.real.txt", sep=""), append=FALSE, sep="\t", col.names=TRUE, row.names=FALSE)
                write.table(all.data, file=paste(mutant, ".all.data.txt", sep=""), append=FALSE, sep="\t", col.names=TRUE, row.names=FALSE)
        
    }   # next mut.i

  } else{ # end if (run.burstsize.regression == TRUE)
    print("Note: not running time vs burst size regresssion. Instead reading results from file.")
  }
```

```
## [1] "Note: not running time vs burst size regresssion. Instead reading results from file."
```

```
  #  ===== Read in and plot ====
  
  burst.size.m <- as.data.frame(matrix(nrow=n.muts, ncol=3))
  colnames(burst.size.m) <- c("slope", "intercpet", "sigma")
  
  for (mut.i in 1:n.muts){
    mutant <- mutant.v[mut.i]
    setwd(bustsize.path)
    burst.size.time.pt.ests <- read.table(file=paste(mutant, ".slope.int.sig.pt.ests.txt", sep=""), header=TRUE)
    burst.size.m[mut.i,] <- burst.size.time.pt.ests
    alpha.hat <- burst.size.time.pt.ests$slope
    mu.hat <- burst.size.time.pt.ests$intercpet
    sig.hat <- burst.size.time.pt.ests$sigma
    alph.v <- read.table(file=paste(mutant, ".slope.v.txt", sep=""), header=TRUE)
      mu.v <- read.table(file=paste(mutant, ".intercept.v.txt", sep=""), header=TRUE)
      sig.v <- read.table(file=paste(mutant, ".sigma.v.txt", sep=""), header=TRUE)
      
      
    
    if (mut.i == 1){
      

      print(paste("EM Convergence Plots for ", mutant, ". Other mutants similarily converge by step 300."))

      plot(alph.v[,1], ylab=expression(paste(hat(alpha))), xlab="EM-Step", type="l", main=mutant, ylim=c(min(alph.v), max(alph.v)*1.3))
      text(100, alph.v[n.em.iterations,1] - ((alph.v[n.em.iterations,1] - alph.v[1,1])/2), label=bquote(paste(hat(alpha)==.(alph.v[n.em.iterations,1]))))
      
      plot(mu.v[,1], ylab=expression(paste(hat(mu))), xlab="EM-Step", type="l", main="", ylim=c(min(mu.v[,1])*0.7, max(mu.v[,1])*1.3))
        text(250, mu.v[n.em.iterations,1] - ((mu.v[n.em.iterations,1] - mu.v[1,1])/2), label=bquote(paste(hat(mu)==.(mu.v[n.em.iterations,1]))))
    
        plot(sig.v[,1], ylab=expression(paste(hat(sigma))), xlab="EM-Step", type="l", main="", ylim=c(min(sig.v[,1])*0.7, max(sig.v[,1])*1.3))
      text(350, sig.v[n.em.iterations,1] - ((sig.v[n.em.iterations,1] - sig.v[1,1])/2), label=bquote(paste(hat(sig)==.(sig.v[n.em.iterations,1]))))
      
      setwd(lambdat0.path)
      mut.pt.est <- read.table(file=paste(mutant, ".lambda.t0.est.txt", sep=""), header=T, sep="\t")
      t.0.hat <- mut.pt.est$t.0.hat
      lambda.hat <- mut.pt.est$lambda.hat
      
      print("Illustration of regression on WT. First plot shows raw count vs time. Burst size vs time relationship (dashed line) is not intuitive from data.")
      mutant <- mutant.v[mut.i]
      setwd(bustsize.path)
      EX.real <- read.table(file=paste(mutant, ".E.X.real.txt", sep=""), header=TRUE)
      all.data <- read.table(file=paste(mutant, ".all.data.txt", sep=""), header=TRUE)
      burst.wells <- which(abs(as.numeric(all.data$burst))>burst.cutoff)   # note use of absolute value so overgrown plates are included as burst wells
        burst.times <- as.numeric(all.data$time[burst.wells])
        max.burst <- max(burst.times)
        burst.wells <- which(as.numeric(all.data$burst)>burst.cutoff)
      bursts <- as.numeric(all.data$burst[burst.wells]) 
      
      plot(x= all.data$time[burst.wells], y=all.data$burst[burst.wells], ylim=c(0, max(as.numeric(all.data$burst))), xlim=c(0,30), xlab="Time", ylab="Observed Count", main="Burst count vs sampled time plus estimated burst size vs time relationship (dashed line)", cex.main=0.8)
      rescaled.int <- mu.hat-(t.0.hat*alpha.hat)
      pt1 <- c(t.0.hat, mu.hat)
      pt2 <- c(35, 35*alpha.hat + rescaled.int)
      points(x=c(pt1[1], pt2[1]), y=c(pt1[2], pt2[2]), type="l", lty="dashed")
      
      #abline(a=mu.hat-(t.0.hat*alpha.hat), b=alpha.hat)

      print("In this second plot, the same burst data is plotted as a function of the expectd sum of burst times obtained from EM algorithm. Now the relationship is clear.")
      plot(EX.real[,500], bursts, xlab="E[sum of burst times, X] (after t0)", ylab="Observed Count", main="Relationship b/t E[sum of burst times, X] and observed burst size with regression line (dashed)", cex.main=0.8)
    abline(mu.hat, alpha.hat, lty="dashed")
    
        print("Same plot shifted right by t0 just to see it on the experimental time scale.")
        plot(EX.real[,500] + t.0.hat, bursts, xlab="E[sum of burst times, X]", ylab="Observed Count", main="Relationship b/t E[sum of burst times, X] and observed burst size plus regression line (dashed)", cex.main=0.8, xlim=c(0,30))
        points(x=c(pt1[1], pt2[1]), y=c(pt1[2], pt2[2]), type="l", lty="dashed")
        

    }
  }
```

```
## [1] "EM Convergence Plots for  WT . Other mutants similarily converge by step 300."
```

```
## [1] "Illustration of regression on WT. First plot shows raw count vs time. Burst size vs time relationship (dashed line) is not intuitive from data."
```

```
## [1] "In this second plot, the same burst data is plotted as a function of the expectd sum of burst times obtained from EM algorithm. Now the relationship is clear."
```

```
## [1] "Same plot shifted right by t0 just to see it on the experimental time scale."
```

```
  colnames(burst.size.m) <- c("alpha.slope", "mu.intercept", "sigma")
  rownames(burst.size.m) <- mutant.v
  print(burst.size.m)
```

```
##       alpha.slope mu.intercept     sigma
## WT      16.668425    28.417193 19.929980
## m.319   19.325386    12.574291  3.222006
## m.321   11.339277    13.878410  7.388327
## m.323   12.372254    14.830017  7.227270
## m.324    8.737669    27.547092 13.032173
## m.5     26.694949    34.567154 17.489247
## m.6     31.632775     7.956547  7.229244
## m.4B    25.081400    16.713661  6.648224
```
