# Supplementary material for: Genetically Determined Variation in Lysis Time Variance in the Bacteriophage φX174
Source: G3 (Bethesda). 2016 Feb 22;6(4):939–55. doi: 10.1534/g3.115.024075 (PMC4825663; doi:10.1534/g3.115.024075)
Supplement: Supporting Information [file supp_g3.115.024075_G3_2015_024075-SI.zip › Lysis_Time_RMarkdown/Raw Data (R-ready, txt)/READ ME.txt]

READ ME

i. File directory
ii. Data format
iii. Special notes

i. File directory
Data from the single-phage assay for each clone is included in the following eight files:
mut319:	319_Data.txt
mut321:	321_Data.txt
mut323:	323_Data.txt
mut324:	324_Data.txt
pos4B:	4B_Data.txt
pos5:		5_Data.txt
pos6:		6_Data.txt
WT:		WT_data.txt

ii. Data format
The first column of each of these tab-delimited files states the time at which each sample was taken (in minutes after the initiation of infection, see paper text, EXPERIMENTAL METHODS).

Each column to the right represents a different instance of the assay performed for a given clone. The date of each instance is provided in "Month_Day_Year" format in the field heading for each column. Items in each column represent the number of particle forming units (PFUs) detected as plaques when the contents of a well were sampled at the indicated time and incubated at 37 degrees C for at least four hours.

iii. Special notes
Five of the time-points in the pos4B data set are repeating decimals. In our analysis, we rounded these times to the nearest tenth of a minute.
